# Enzymatic Hydroxylation of Aliphatic C–H Bonds by a Mn/Fe Cofactor

**DOI:** 10.1021/jacs.3c03419

**Published:** 2023-07-20

**Authors:** Magan M. Powell, Guodong Rao, R. David Britt, Jonathan Rittle

**Affiliations:** † Department of Chemistry, University of California, Berkeley, Berkeley, California 94720, United States; ‡ Department of Chemistry, University of California, Davis, Davis, California 95616, United States

## Abstract

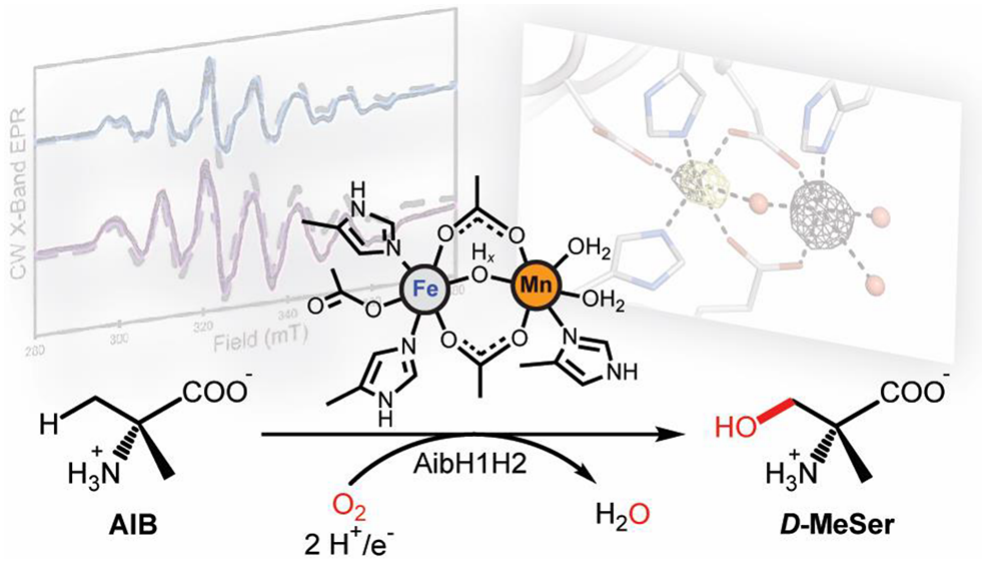

The aerobic oxidation of carbon–hydrogen (C–H) bonds in biology is currently known to be accomplished by a limited set of cofactors that typically include heme, nonheme iron, and copper. While manganese cofactors perform difficult oxidation reactions, including water oxidation within Photosystem II, they are generally not known to be used for C–H bond activation, and those that do catalyze this important reaction display limited intrinsic reactivity. Here we report that the 2-aminoisobutyric acid hydroxylase from *Rhodococcus wratislaviensis*, AibH1H2, requires manganese to functionalize a strong, aliphatic C–H bond (BDE = 100 kcal/mol). Structural and spectroscopic studies of this enzyme reveal a redox-active, heterobimetallic manganese–iron active site at the locus of O_2_ activation and substrate coordination. This result expands the known reactivity of biological manganese–iron cofactors, which was previously restricted to single-electron transfer or stoichiometric protein oxidation. Furthermore, the AibH1H2 cofactor is supported by a protein fold distinct from typical bimetallic oxygenases, and bioinformatic analyses identify related proteins abundant in microorganisms. This suggests that many uncharacterized monooxygenases may similarly require manganese to perform oxidative biochemical tasks.

## Introduction

The biochemistry of manganese is intimately linked to dioxygen (O_2_). Nearly all of the O_2_ in the atmosphere was generated via water oxidation at a manganese-containing cofactor in Photosystem II.^1,2^ This enzyme was largely responsible for the Great Oxygenation Event that enabled the emergence of multicellular life.^3^ Aerobic respiration in these organisms inadvertently generates reactive oxygen species, such as superoxide and peroxide, that are often neutralized by Mn-dependent enzymes. Most mitochondria express a Mn-dependent form of superoxide dismutase to safeguard core respiratory enzymes,^4^ and bacteria lacking access to heme cofactors detoxify hydrogen peroxide with Mn-dependent catalases.^5^

Given these roles, it is perhaps surprising that very few Mn enzymes are known to use O_2_ to functionalize other substrates. A few ring-cleaving dioxygenases coordinate O_2_ at a Mn site before its insertion into catechol substrates,^6,7^ and some fungi employ manganese lipoxygenase to generate reactive lipid metabolites.^8,9^ In these latter enzymes, a mononuclear Mn center selectively activates a weak C–H bond (BDE_CH_ = 77 kcal/mol) to generate a radical intermediate that is captured by molecular O_2_. Stronger C–H bonds are not activated. Many bacterial ribonucleotide reductases require a peripheral Mn_2_ or Mn/Fe site to activate superoxide or O_2_, respectively, but these metals do not directly participate in the bond-breaking/making reactions occurring at the ribose substrate.^10−14^ Besides these enzymes, there are only two other Mn-containing proteins that use O_2_ to mediate C–H bond activation processes in a *stoichiometric* fashion: ribonucleotide reductase R2-like ligand-binding oxidase (R2lox) and the *Chlamydia* protein associating with death domains (CADD).^15,16^

Of course, many non-Mn enzymes use O_2_ to activate strong, aliphatic C–H bonds.^17−20^ Nature has evolved a large and diverse repertoire of hydroxylases for xenobiotic detoxification,^17^ regulation of cellular function,^21^ and the construction of bioactive natural products.^20^ Yet the embedded cofactors that carry out these chemical processes are exclusive: heme, nonheme iron, flavin, and copper centers are the only biological cofactors known to functionalize aliphatic C–H bonds.^17,22−25^ Accordingly, our foundational understanding of aerobic C–H bond activation stems from the chemical mechanisms operative for this limited set of cofactors.

**Figure 1:**
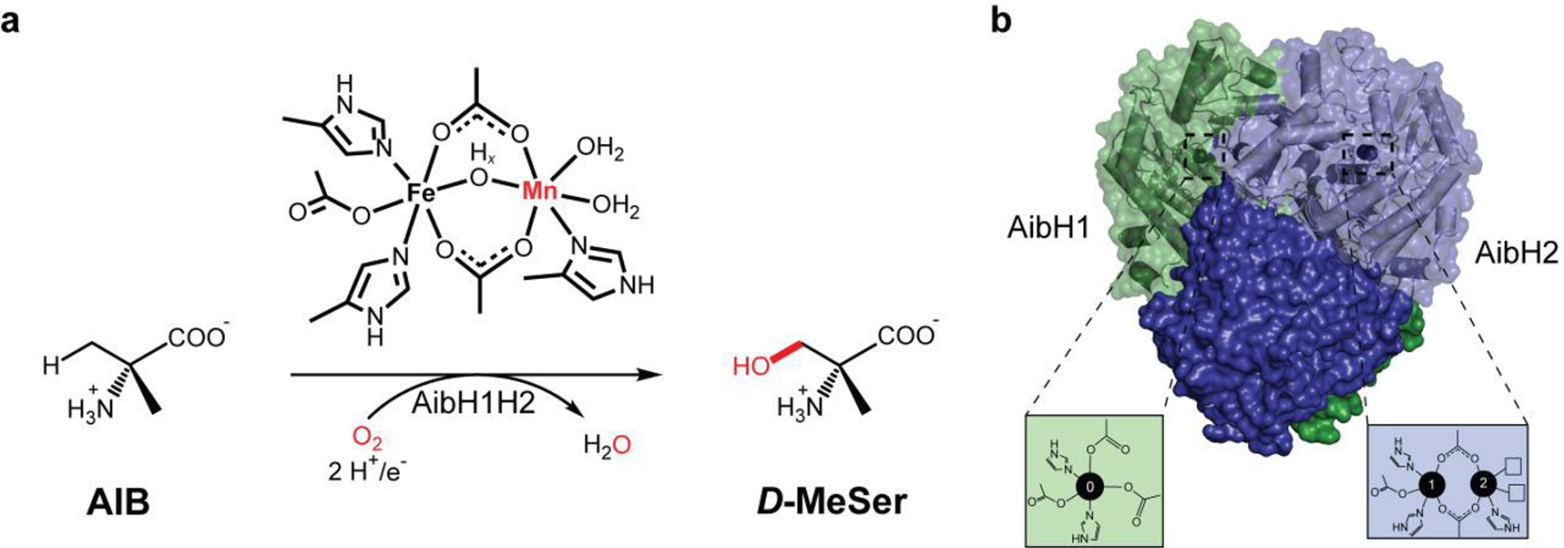
(A) The enzymatic reaction catalyzed by AibH1H2^30^ and its active site cofactor described in this work. (B) Cartoon and surface representation of the AibH1H2 (αβ)_2_ heterotetramer, made up of AibH1 (green) and AibH2 (blue) subunits alongside their respective metal binding sites.

The absence of natural Mn hydroxylases implies either that this metal ion is not competent to mediate hydroxylation reactions or that such enzymes have eluded discovery. The former explanation is challenged by the numerous *synthetic* catalysts that harness manganese to mediate challenging oxidative chemical reactions including olefin epoxidation, aliphatic C–H bond hydroxylation, and halogenation.^26−29^ In this report, we demonstrate that the activity of a *Rhodococcus* hydroxylase exhibits a strict dependence on manganese and specifically utilizes an unusual heterometallic Mn/Fe cofactor to effect the catalytic functionalization of an unactivated, primary C–H bond (Figure 1a).

## Results and Discussion

### Establishing the Metal Dependence on AibH1H2 Enzymatic Activity

Metabolism of 2-aminoisobutyric acid (AIB) in *Rhodococcus wratislaviensis* proceeds via the initial, selective hydroxylation of the pro-(*R*) methyl group by the monooxygenase AibH1H2 to furnish α-methyl-d-serine (d-MeSer).^30^ Previously, recombinant expression of AibH1H2 in *Escherichia coli* enabled its structural characterization and revealed distinct mono- and dinuclear metal binding sites housed within the AibH1 and AibH2 protein subunits, respectively (Figure 1b).^30^ Metal analyses performed on these samples suggested the presence of iron and zinc ions, leading the authors to propose a structural mononuclear zinc site in AibH1 and a diiron site in AibH2 that serves as a locus for hydroxylation. Consistent with the latter proposal, many diiron hydroxylases are known.^20,23,31^ The AibH2 subunit is also structurally related to the dinuclear hydroxylase PtmU3, which displayed superior enzymatic activity in the presence of exogenously supplied iron ions.^32^ But the activity of recombinant AibH1H2 derived from *E. coli* was not determined. Instead, conversion of AIB to d-MeSer was only observed via whole-cell recombinant expression of AibH1H2 in *Rhodococcus erythropolis.* These discrepancies in enzyme preparation motivated our independent determination of the active metalated form of AibH1H2.

**Figure 2:**
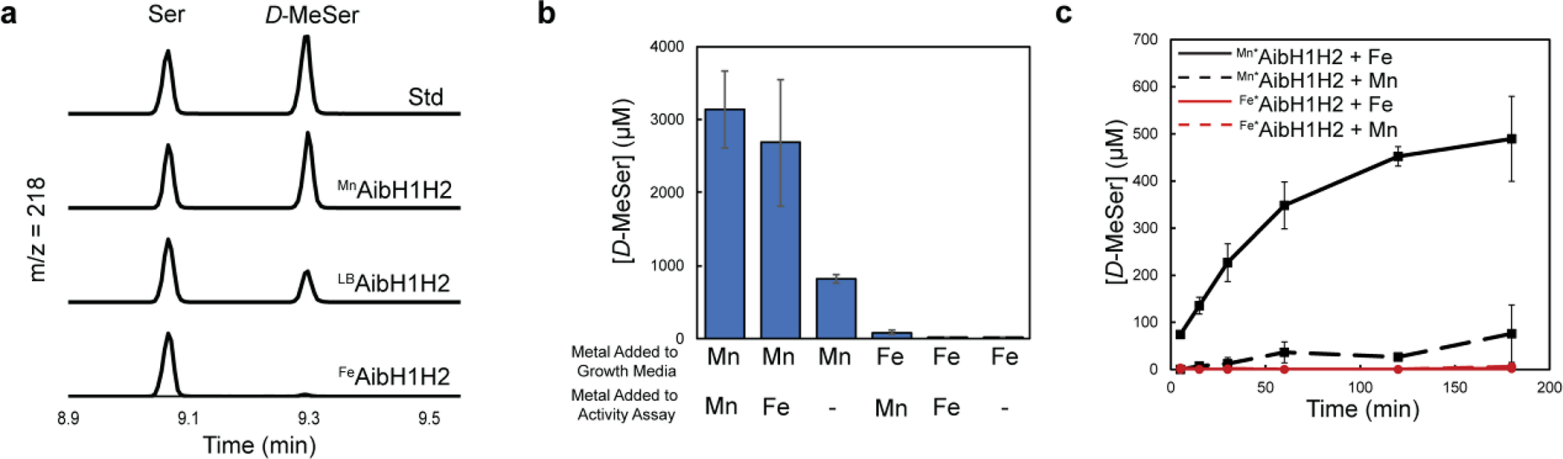
Catalytic activity of 25 μM AibH1H2 under different metalation conditions. (A) GC-MS chromatograms filtered at *m*/*z* = 218 (Figures S2 and S3) of representative catalytic activity assays of AibH1H2 under different growth conditions with no additional metal added during the activity assay. (B) Catalytic activity of ^Fe^AibH1H2 and ^Mn^AibH1H2 (Table 1, entries 1 and 3, respectively) with one additional equivalent of metal or an equal volume of water (denoted with “–”), added to the reaction mixture. (C) Time-dependent generation of d-MeSer by ^Mn*^AibH1H2 (black traces, Table 1 entry 4) and ^Fe*^AibH1H2 (red traces, Table 1 entry 5) with one additional equivalent of Mn (dashed trace) or Fe (solid trace) added to the reaction mixture.

We expressed AibH1H2 in *E. coli* grown in M9 minimal medium supplemented with excess Fe^II^ (Scheme S1), and subsequent purification furnished protein samples (^Fe^AibH1H2) containing ∼3 equiv of Fe per AibH1H2 heterodimer and minimal heterometal content (Table 1, entry 1). Fe occupancy in both the mononuclear (Site 0) and dinuclear (Sites 1 and 2) metal sites was evident by inspection of the anomalous dispersion maps obtained from X-ray diffraction experiments performed on single crystals of ^Fe^AibH1H2 (Figure S1). AibH1H2 had no detectable activity upon the exclusive incorporation of iron ions. Initial assays to determine the enzymatic activity were performed by mixing ^Fe^AibH1H2 with the AIB substrate and sodium ascorbate as a sacrificial reductant under aerobic conditions. Subsequent workup and gas chromatography–mass spectrometry (GC-MS) analyses of these solutions revealed minimal d-MeSer production (Figure 2a). Specifically, an ion fragment at *m*/*z* = 218, diagnostic for silylated d*-*MeSer, is absent at the expected retention time (Experimental Methods, Figures S2 and S3). To probe whether the absence of monooxygenase activity was caused by the choice of sacrificial reductant, other commonly utilized reducing systems (e.g., NADH/Phenazine, sodium dithionite, and alpha-ketoglutarate) were explored, but none proved competent to produce measurable quantities of d-MeSer with ^Fe^AibH1H2 (Figure S4). These results suggested that the active form of AibH1H2 could not be produced under routine expression and purification conditions typically used for nonheme iron enzymes.

**Table 1:** Metal Content and Reactivity of Different AibH1H2 Preparations^a^

	metal/heterodimer				
	Fe	Mn	Ni	Zn	[d-MeSer]/[AibH1H2]^b^
^Fe^AibH1H2	2.8 ± 0.1	0.08 ± 0.04	0.03 ± 0.03	0^c^	<2^d^
^LB^AibH1H2	1.67 ± 0.07	1.05 ± 0.01	0.055 ± 0.003	0.131 ± 0.004	33 ± 1
^Mn^AibH1H2	0.4 ± 0.1	2.2 ± 0.4	0.03 ± 0.07	0	110 ± 30
^Mn*^AibH1H2	0	2.4 ± 0.3	0	0	50 ± 10
^Fe*^AibH1 H2	1.8 ± 0.2	0	0	0	<2
Mn/Fe Crystal	0.0	1.9	1.3^e^	0.0	30
EPR	0.3	1.5	0.0	0.1	73

a Data for entries 1–5 represent the average of at least three technical replicates. Error is the standard deviation. Entries 6 and 7 represent the specific batch used for the application indicated. Error of the ICP-OES instrument is less than 0.01 ppm.

b Catalytic activity assay performed with the addition of one equivalent of Fe^II^ relative to AibH1H2 (25 μM) in the reaction mixture.

c Values less than the limit of detection of the ICP-OES instrument (0.01 ppm) are reported as zero.

d Values less than 2 are below the minimum point on the calibration curve for GC-MS analysis of catalytic activity.

e High Ni content is likely due to contamination from the Ni-NTA column during protein purification.

The hydroxylation of AIB is dependent upon the incorporation of manganese into AibH1H2. The expression of AibH1H2 in rich medium (lysogeny broth) furnished samples (^LB^AibH1H2) that contained ∼1.7 equiv of Fe and ∼1 equiv of Mn per AibH1H2 heterodimer (Table 1, entry 2). The incorporation of tightly bound manganese ions was evident upon inspection of the electron paramagnetic resonance (EPR) spectra of exhaustively dialyzed samples (Figure S5). Unlike the case for ^Fe^AibH1H2, enzymatic assays employing ^LB^AibH1H2 yielded substantial quantities of d-MeSer (33 equiv/AibH1H2). Since the protein structures of these two samples were found to be identical (RMSD = 0.34 Å over all atoms; Figure S1c), our findings suggested an apparent requirement for one or more Mn ions in AibH1H2. This hypothesis was confirmed upon expression of AibH1H2 in M9 minimal medium supplemented with excess Mn^II^ instead of Fe^II^ to afford samples (^Mn^AibH1H2) that displayed 3-fold higher specific hydroxylase activity and likewise a higher Mn content (Table 1, entry 3) relative to ^LB^AibH1H2.

The proposal of a Mn-dependent monooxygenase is unexpected because previous reports of Mn-dependent hydroxylation have been discredited due to Fe contamination.^12,33,34^ Similarly in our case, the presence of Fe and other contaminating heterometals in the catalytically active as-isolated preparations of AibH1H2 obscured the identity of the most active cofactor (Figure 2b). We thus subjected these protein samples to mild metal chelation protocols, which decreased overall activity (Figure S6) but standardized the metal content. The resultant samples contained either ∼2 equiv of Mn (^Mn*^AibH1H2) or ∼2 equiv of Fe (^Fe*^AibH1H2) and minimal contamination with other metals (Table 1, entries 4 and 5). These partially metalated samples exhibited minimal enzymatic activity (Figure S7). Assays were then performed in the presence of an additional 1 equiv of Fe^II^ or Mn^II^ in order to vary the identity of the third metal site *in situ*. Hydroxylation of AIB was prominently mediated by ^Mn*^AibH1H2 upon addition of 1 equiv of Fe^II^ (Figure 2c, Figure S7). No reaction of the ^Fe*^AibH1H2 protein, regardless of metal addition, yielded product above the limit of detection of the analysis, and similarly low activity was observed following exposure of ^Mn*^AibH1H2 to an additional 1 equiv of Mn^II^. Collectively, these results indicated that the incorporation of two manganese ions and one iron ion afforded the most active form of AibH1H2.

### Characterization of the AibH1H2 Cofactors

**Figure 3:**
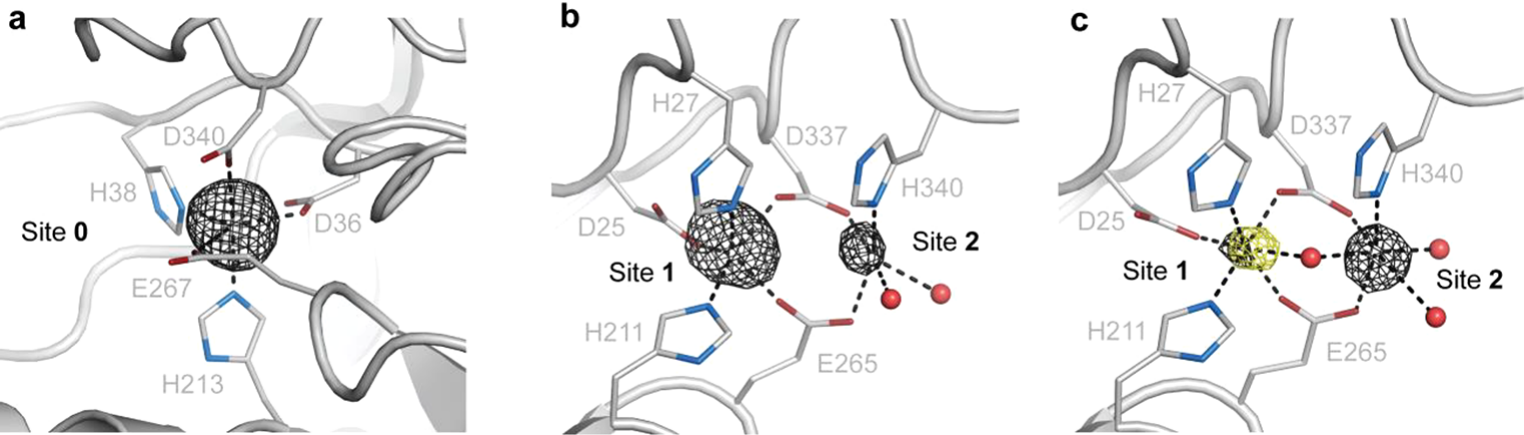
Characterization of the AibH2 Mn/Fe cofactor (PDB: 8FUN). Cartoon representations of the ^Mn*^AibH1H2 protein structure at Site 0 (A), View 1 (B), and View 2 (C) of the dinuclear cofactor are shown overlaid with the anomalous density difference map at the Mn K-edge (black) and the dual-wavelength isomorphous anomalous density difference map near the Fe K-edge (yellow), each contoured at 5.0σ.

To determine the locations of these metal ions, crystallographic studies were performed on active preparations (Table 1, entry 6) of AibH1H2. A single crystal grown from a sample of ^Mn*^AibH1H2 premixed with 2 molar equiv of Fe^II^ per AibH1H2 heterodimer was examined by multiwavelength X-ray crystallography (PDB 8FUN). The unique locations of metal ions were determined by analysis of the anomalous X-ray dispersion collected at either the Mn K-edge or an anomalous isomorphous difference map stemming from diffraction data collected above and below the Fe K-edge. Two AibH1H2 heterodimers are found in the asymmetric unit. Both AibH1 subunits display strong anomalous scattering at the Mn K-edge above background levels (Table S1) and lack observable density at the Fe K-edge difference map (Figure S8) at Site 0, supporting exclusive Mn binding at these sites (Figure 3a). In contrast, the dinuclear sites in AibH2 appear in two distinct metalated forms. View 1 of AibH2 (Figure 3b) contains a dimanganese site with a fully occupied Site 1 metal and a partially occupied (∼60%) Site 2 metal. View 2 of AibH2 contains two fully occupied sites with predominant Fe occupancy in Site 1 and exclusive manganese occupancy in the solvent-exposed Site 2 (Figure 3c). Since this latter view represents the only location of observable Fe content throughout the structure, we hypothesize that the bimetallic arrangement of View 2 represents the correctly metalated form of AibH1H2 that participates in substrate hydroxylation.

**Scheme 1:**
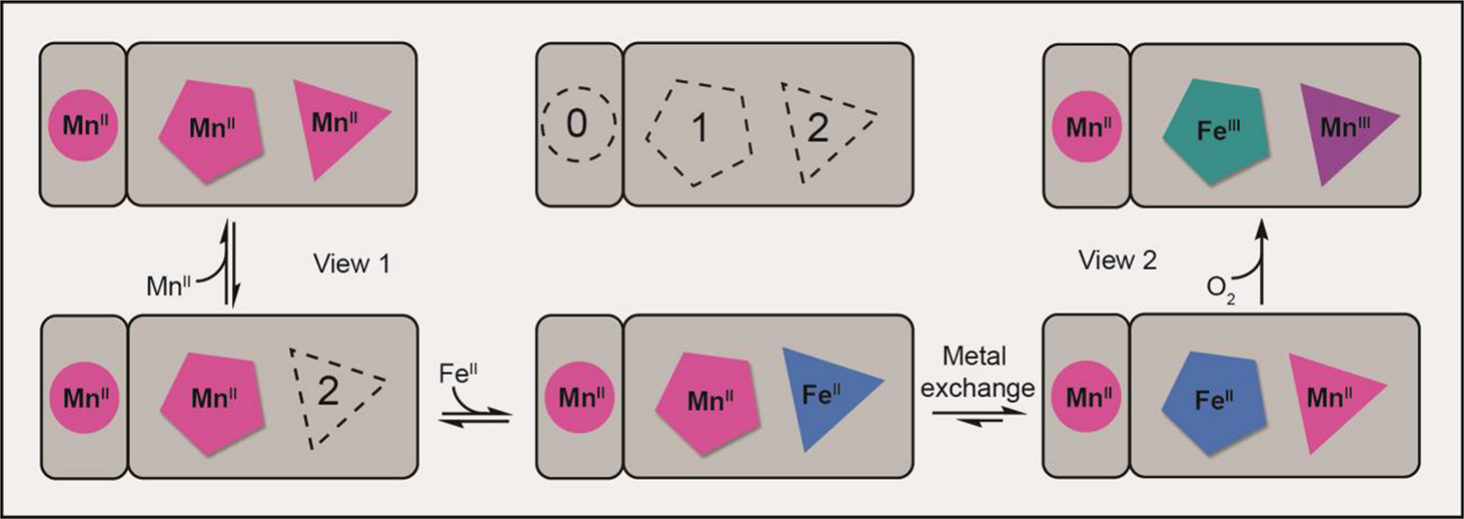
Proposed Metalation Scheme of AibH1H2 Site 0 is depicted with a Mn^II^ ion to reflect the crystallographic data (PDB: 8FUN), but the physiological identity of the metal is unknown.

These crystallographic snapshots allowed us to formulate a hypothesis for the cofactor assembly in AibH1H2. In the presence of high Mn^II^ concentrations, this ion will bind to Site 0 and to one or both sites of the AibH2 dinuclear cofactor (Scheme 1). Fe^II^ ions are expected to displace Mn^II^ ions in a mixed Fe^II^/Mn^II^ environment as they form stronger metal–ligand bonds.^35^ However, slow metal dissociation kinetics may prevent observable metal exchange, which is a possible explanation for the substitutionally inert nature of the mononuclear Mn^II^ site in AibH1 upon exposure to Fe^II^. Given that Site 2 of AibH2 is more solvent exposed and labile (View 1, Figure 3b), a kinetic argument would suggest that Fe^II^ would first bind at Site 2. However, under thermodynamic control and *limiting iron*, this ion is expected to bind to the most tightly chelating site. In this scenario, Site 1 is the expected and observed position for Fe^II^ binding, as the coordination environments of Sites 1 and 2 contain five and three amino-acid-derived ligands, respectively. Thus, we propose that Fe^II^ might initially bind at Site 2, but eventually exchanges to the thermodynamically preferred Site 1 position. Air oxidation of AibH1H2 furnishes trivalent metal ions in its resting state (*vide infra*) that may serve to lock the ions into their observed positions (Scheme 1). This proposed mechanism is similar to that proposed for the Mn/Fe cofactor in class Ic ribonucleotide reductase but distinct from that of R2lox.^10,36−38^

In cellular contexts, the concentrations of free Mn^II^ and Fe^II^ present in the cytoplasm during protein synthesis will influence the outcome of the final metalated state of AibH1H2. These metal ion concentrations can vary widely and are dependent on numerous factors. For example, *E. coli* maintains ∼100 μM Fe^II^ and ∼10 μM Mn^II^ in its cytoplasm, whereas *Bacillus subtilis* is known to possess a 10-fold higher concentration of Mn^II^ under similar growth conditions.^12^ Although the free ion concentrations are likely lower than the values listed above, these numbers portray the variability in the metal ion content in these organisms. We speculate that *R. wratislaviensis* must possess a relatively high Mn^II^ concentration to allow proper construction of the active heterobimetallic cofactor in AibH1H2. While the metallome of *R. wratislaviensis* has yet to be experimentally determined, it is noteworthy that the ribonucleotide reductases present in its proteome are all Mn-dependent class Ib, as determined by the co-localization of the *nrdI* gene, an established indictor of class Ib RNR (Figure S9).^14,39−41^ This supports the hypothesis that *R. wratislaviensis* maintains a high concentration of free cytoplasmic Mn^II^. Alternatively, endogenous chaperone proteins^42^ may selectively deliver manganese ions to the pertinent metal sites during protein synthesis, although experimental data in support of either proposal are not presently available.

Having established a plausible metal content for active AibH1H2 preparations, we set out to determine the fundamental biochemical characteristics of this hydroxylation reaction. Monooxygenase activity of ^Mn^AibH1H2 in the presence of 1 equiv of Fe^II^ was firmly established via performance of analogous assays under an atmosphere of ^18^O_2,_ which revealed the formation of ^18^O-labeled d-MeSer as evidenced by a +2 *m*/*z* shift in its diagnostic ion fragment (Figure S10). This experiment establishes that one atom of molecular oxygen is incorporated into MeSer by AibH1H2. Preliminary attempts to ascertain Michaelis–Menten parameters of AIB hydroxylation curiously intimate a large *K*_M_ (>100 mM) suggestive of weak substrate coordination under our *in vitro* reaction conditions (Figure S11). Given the prior characterization of the other genes in the AibH1H2 operon, AIB appears to be the native substrate despite the low binding affinity.^30^ An associated Rieske protein and flavoenzyme, AibG and AibF, respectively, were assigned as the native electron transport chain between NADH and AibH1H2,^30^ and it is possible that the native protein–protein interactions *in vivo* influence AIB coordination, but this remains to be determined. Finally, the secondary sphere environment surrounding the dinuclear cofactor was found to be critical in enabling the enzymatic reaction to proceed, as evidenced by the behavior of a D342A mutant of AibH2. This aspartate residue hydrogen bonds to one of the terminal aquo ligands of the Site 2 metal ion and, upon substitution to alanine, results in protein with diminished enzymatic activity despite its ability to incorporate a complete repertoire of metal ions (Figure S12).

**Figure 4:**
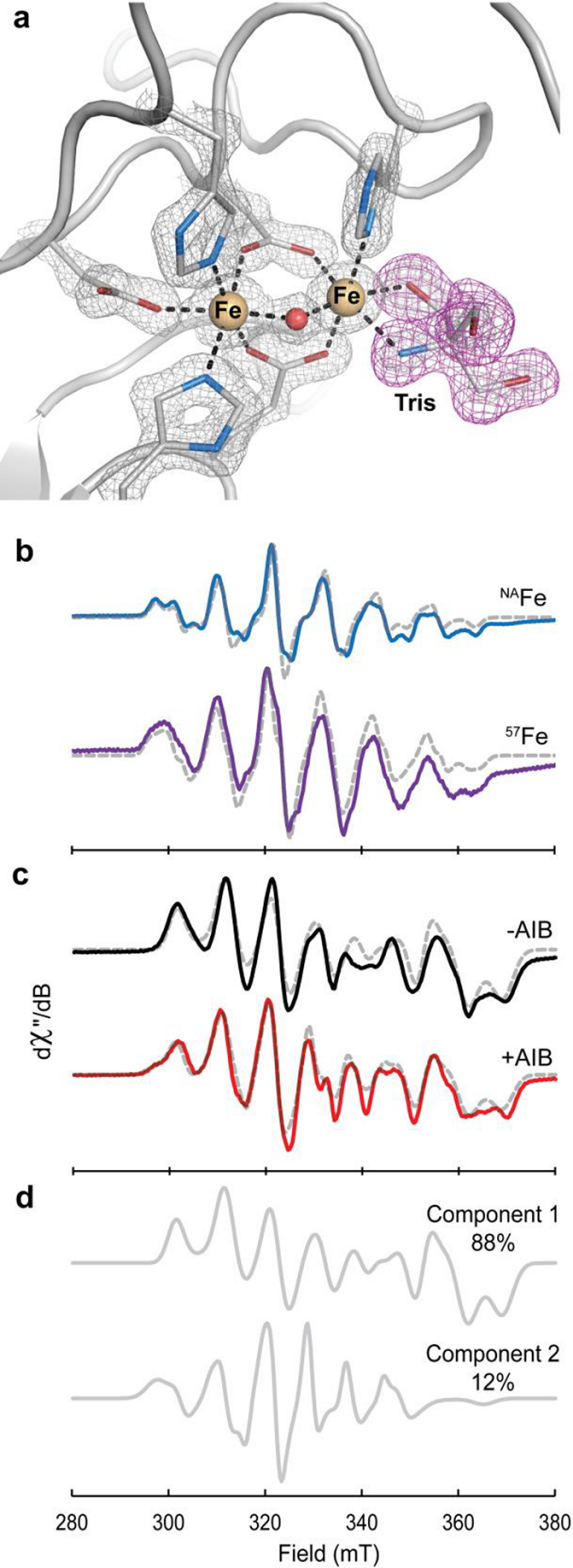
(a) High-resolution crystal structure of ^Fe^AibH1H2 crystallized in the presence of Tris. 2*F*_o_ – *F*_c_ electron density is contoured at 2.5σ and shown in gray mesh. (b) Continuous-wave X-band EPR spectra of ^Mn^AibH1H2 in 1 M Tris with one additional equivalent of natural abundance Fe (top, blue) or ^57^Fe (bottom, purple) collected at 15 K and 20 mW power. (c) EPR spectra of ^Mn^AibH1H2 in CHES without (top, black) and with (bottom, red) 100 mM AIB taken at 12 K and 63.25 mW power. Simulations are shown in gray dashed lines. (d) The extracted components used to simulate the CHES/AIB sample. Component 1 has identical parameters to the 20 mM CHES samples without AIB, and Component 2 represents a new species. Refer to Table S2 for simulated spin Hamiltonian parameters. Tris = tris(hydroxymethyl)aminomethane, CHES = *N*-cyclohexyl-2-aminoethanesulfonic acid.

Available crystallographic and analytical data strongly support the presence of Mn and Mn/Fe cofactors in the active form of AibH1H2, but the locus of redox activity necessary for the activation of O_2_ and substrate remains to be determined. Unlike Site 0, the AibH2 dinuclear cofactor is solvent-exposed. A 1.5 Å X-ray crystal structure of ^Fe^AibH1H2 grown in the presence of Tris (Figure 4a, PDB 8FUM) illustrates that exogenous small molecules can access and coordinate to the Site 2 metal, supporting a functional role for the dinuclear cofactor in AibH2. EPR spectroscopy was used to recapitulate this ligand-bound structure in solution and determine the reactivity of Mn- and Fe-metalated AibH1H2 with O_2_ and small molecules. First, a sample of ^Mn^AibH1H2 (Table 1, entry 7) was combined with 1 equiv of Fe^II^ and Tris and subsequently exposed to air for 1 h. Two distinct paramagnetic species were observed in the resulting EPR spectra of these frozen solutions. An isotropic signal characteristic of a mononuclear *S* = 5/2 manganese ion was predominant at high temperatures and ascribed to a Mn^II^ cofactor in Site 0 and/or adventitiously bound Mn^II^ (Figure S13). This signal could be effectively saturated at high power and low temperature to reveal an unobscured view of the second EPR-active species. This latter signal (Figure 4b) contained well-resolved features that are noticeably perturbed when isotopically enriched ^57^Fe was incorporated into the sample. These spectra are similar to the *S*_TOT_ = 1/2, antiferromagnetically coupled Mn(III)–Fe(III) forms of *C*. *trachomatis* ribonucleotide reductase and R2lox.^13,43,44^ Indeed, spectral simulations revealed ***g***- (2.03 2.03 2.02) and ^55^Mn hyperfine (***A***_Mn_ = [300 250 380] MHz) tensors consistent with the respective spin and oxidation state assignments. The isotropic ^57^Fe hyperfine tensor (***A***_Fe_ = [−70 −70 −70] MHz) further supported the assignment of a high-spin Fe(III) oxidation state. These experiments confirm that the AibH2 cofactor is redox-active and readily oxidized by O_2_ to generate a Mn(III)–Fe(III) redox state.

Since direct substrate coordination to a metal center is observed in some diiron oxygenases,^45,46^ we explored the possibility of AIB substrate coordination to the Mn/Fe cofactor in AibH2. The EPR spectrum of AibH1H2 prepared in the absence of coordinating small molecules (Figure 4c, top) was markedly distinct from that found in the presence of Tris. Most notably, low-field features are lost (<300 mT) and new features emerge at higher fields (∼370 mT), and these spectra hence required a distinct set of Hamiltonian parameters for their effective simulation (Table S2). Inclusion of 100 mM AIB to similarly prepared samples resulted in a spectrum displaying subtle but reproducible perturbations (Figure 4c, bottom). This EPR signal could be simulated only as a composite of two species with a new 12% component ascribed to an AIB-bound form (Figure S14). The parameters of this component resemble those of AibH1H2 in the presence of Tris, suggesting that both AIB and Tris perturbed the spin center in similar manners.^44^ We propose that AIB is bound either via direct coordination to the Site 2 metal (i.e., Mn) or in a noncovalent manner that substantially alters the geometry and/or protonation state of one or more coordinated ligands. Together, the redox-active core and coordination of AIB at the dinuclear site strongly support the direct involvement of the heterobimetallic Mn/Fe cofactor in substrate hydroxylation by AibH1H2.

The data collected in this study allow us to make mechanistic hypotheses about the roles of the Mn/Fe cofactor in AibH1H2. The Mn(III)–Fe(III) redox form characterized herein appears to represent a dominant state of AibH1H2 under aerobic conditions. This state does not directly convert AIB to d-MeSer (Figure S4) and appears to require reductive activation for detectable substrate conversion. Presumably, our *in vitro* reductant, ascorbate, or the biological redox partners (AibG and AibF) mediate the reduction of the Mn(III)–Fe(III) cofactor of AibH1H2 to one or more lower valent states (e.g., Mn(II)–Fe(III) or Mn(II)–Fe(II)) primed for O_2_ binding. Borrowing mechanistic precedence from well-studied nonheme diiron oxygenases, we hypothesize that O_2_ will coordinate to one of these low-valent forms of the cofactor to generate sequential peroxo and dioxo intermediates competent for C–H bond activation^19^ and substrate hydroxylation. Overall, the timing of AIB coordination with respect to O_2_ coordination and activation remains unknown. Since the AIB substrate only effects a marginal perturbation of the EPR features associated with the Mn(III)–Fe(III) state, we speculate that efficient substrate binding may be gated by prior reduction of the cofactor. The Mn(III)–Fe(III) state hence may reflect the enzymatic redox state directly following AIB hydroxylation and d-MeSer release. Alternatively, the minor perturbation in the EPR features upon AIB addition could be due to the apparent low binding affinity under the *in vitro* conditions employed in this study that lack the biological reductase components (*vide supra*). Work to clarify the mechanistic aspects of this unusual enzyme is ongoing in our laboratories.

While the above data suggest that AibH2 is the locus for hydroxylation, the role and physiological metal identity of AibH1 remain elusive. We disfavor Site 0 as the site of AIB hydroxylation owing to its sterically occluded nature (Figure S8b) and instead speculate that it serves as a structural metal to maintain the quaternary protein fold needed for enzymatic activity. First, multiple attempts to disrupt metal binding to Site 0 via substitutions of either metal-binding residues or secondary-sphere residues in AibH1 resulted in insoluble protein (Supporting Information). Second, it is not clear if there is a specific metal requirement for Site 0, which may suggest that it does not serve a functional redox role. For example, while our most active preparations have Mn in Site 0, other preparations, specifically ^LB^AibH1H2, possess only 1 Mn ion and ∼2 Fe ions but are still competent for turnover. Although the precise location of the metals was not determined for this protein preparation, our biochemical data suggest that either (a) one of the Fe ions must occupy Site 0 or (b) there is a mixture of metalated and mismetalated protein if Mn must occupy Site 0 and Site 2. If the former is true, it would suggest that the metal identity of Site 0 is not specific and AibH1 may accept the most abundant metal available in the cellular environment. Third, AibH1-like proteins are not always necessary for hydroxylation, since PtmU3—the only other known hydroxylase stemming from the protein structural superfamily—does not associate with an auxiliary mononuclear protein. Further experiments, however, are necessary to definitively assign the role and metal dependency of Site 0 in AibH1.

### Identification of a Widespread Family of AibH2-like Enzymes

**Figure 5:**
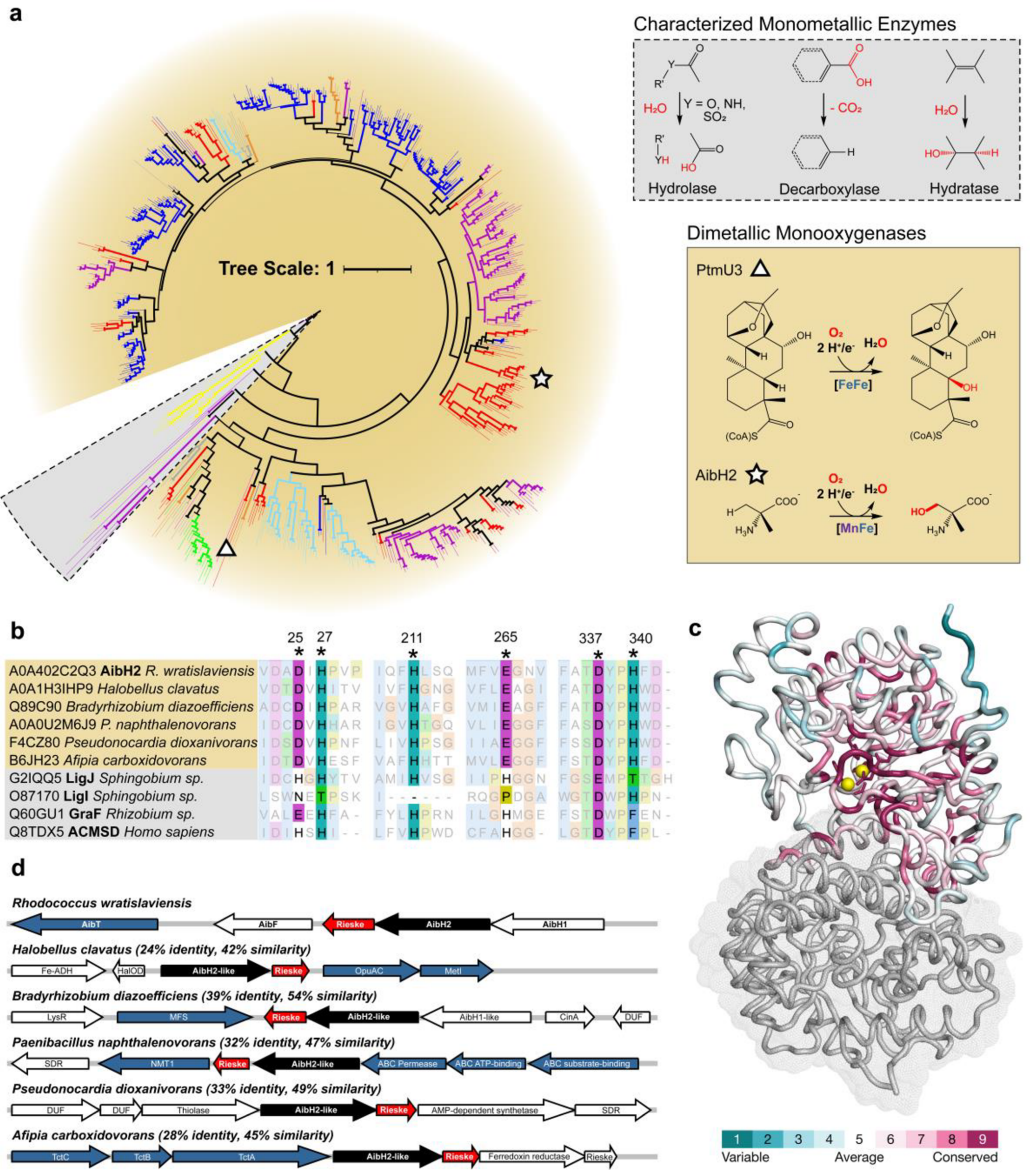
Bioinformatic identification of an uncharacterized family of AibH2-like proteins. (A) Unrooted, neighbor-joining phylogenetic tree of 555 Uniprot Reference Proteome sequences with at least 24% identity to AibH2 and conserved metal-coordinating residues. Biochemically characterized representatives of the PF04909 protein family were included for context. Branch thickness is proportional to the bootstrap values. The branches are colored according to the taxonomic group: red: actinobacteria; magenta: proteobacteria; blue: bacilli; orange: chloroflexi; yellow: eukaryote; teal: haloarchaea; green: cyanobacteria; black: other. The underlying shading reflects the enzymatic reactions (inset) known or expected to be catalyzed by these enzymes. Tree scale, 1.0 amino acid substitution per site. (B) Multiple sequence alignment of representative candidate monooxygenase sequences and characterized monometallic enzymes highlighting the metal binding residues found in AibH2. (C) Ribbon and surface representation of AibH1H2 illustrating the localization of variable (cyan) and conserved (purple) regions of 300 randomly chosen AibH2-like sequences. (D) Genome neighborhood diagrams of AibH2 and AibH2-like proteins. Genes are colored according to an inferred function: black: AibH2-like monooxygenase; red: Rieske-type ferredoxin; blue; small-molecule permease components.

Owing to the paucity of Mn-dependent monooxygenases, we searched for proteins related to AibH2 to determine the biological distribution of enzymes competent to harbor a similar Mn/Fe cofactor. The amino acid sequence of AibH2 is highly divergent from all of the biochemically characterized enzymes of this protein family (PF04909) that more commonly contain monometallic zinc cofactors and catalyze nonredox reactions (Figure 5a).^47−49^ PtmU3, the other established monooxygenase in this protein family, displays 27% sequence identity or 40% similarity to AibH2.^32^ A multiple-sequence alignment of the UniProt reference proteome sequences displaying >24% identity to AibH2 identified 555 proteins containing all six amino acid residues found to coordinate the Mn/Fe cofactor in AibH2 (Figure 5b). All of these proteins are presently uncharacterized but predicted to possess protein folds and active site structures similar to those of AibH2 (Figure 5c). The constitutions of the genomic neighborhoods (Figure 5d) surrounding the corresponding genes are distinct from those of AibH1H2 and collectively contain few co-occurring proteins that could be used to infer their precise biological function(s). However, a conspicuous 90% co-occurrence of an adjacent small Rieske protein and 40% co-occurrence of small-molecule permeases provide support that these operons are engaged in the catabolism of unknown small molecules. In particular, the tight genomic association between the AibH2-like proteins with the Rieske proteins is reminiscent of catabolic monooxygenases (e.g., cytochrome P450, soluble methane monooxygenase), which frequently require a dedicated, endogenous reductase.^17,23^ Accordingly, we postulate that these 555 uncharacterized PF04909 proteins represent members of a new class of monooxygenases that may harbor Mn/Fe cofactors to catalyze as yet unknown hydroxylation reactions. While the metalation state of the expected dinuclear cofactors of these proteins cannot be unambiguously determined from genomic information alone, it is noteworthy that a large portion of these uncharacterized proteins stem from organisms known to display high cytoplasmic manganese concentrations, including members of *Bacilli*,^12^ radiation- and/or desiccation-resistant bacteria,^50,51^ and halophilic microorganisms^52^ Experimental efforts to establish the enzymatic reactivity and cofactor content of these candidate monooxygenases are ongoing in our laboratories.

## Conclusions

The *in vitro* studies of AibH1H2 presented in this work provide the first unambiguous evidence of Mn-dependent monooxygenation in Nature. The available reactivity, crystallographic, and spectroscopic data collectively support a redox-active Mn/Fe cofactor that can activate O_2_ and bind AIB en route to its hydroxylation. The presence of manganese at the key site of AIB coordination (Site 2) emphasizes its critical role in the functionalization of a strong aliphatic C–H bond. Accordingly, this unusual Mn/Fe cofactor exhibits reactivity on par with that of the highly reactive Fe- or Cu-dependent hydroxylase active sites. Our results expand the known roles of manganese in biology and motivate further studies to understand how and where Mn-dependent monooxygenases function.

## Experimental Methods

### General

All chemicals were purchased from commercial suppliers and used directly as received, unless noted otherwise. The plasmid pACYC-GroEL/ES-TF was a gift from Karl Griswold (Addgene plasmid #83923; http://n2t.net/addgene:83923; RRID: Addgene 83923). Metal analysis was conducted in the Microanalytical Facility in the College of Chemistry at the University of California, Berkeley, by using a PerkinElmer ICP Optima 7000 DV spectrometer. All degassed chemicals were rendered anaerobic by equilibration overnight in a Coy anaerobic chamber equipped with a 1.5–2.5% H_2_/N_2_ environment and a palladium catalyst. All aqueous solutions were prepared using water purified by a Milli-Q Academic water purifier and exhibited a conductivity of 18.2 MΩ. Genetic sequencing was performed at the University of California, Berkeley, DNA Sequencing Facility. Large-scale (>0.5 L) bacterial cultures were grown using a LEX-48 bioreactor (Epiphyte3). An Agilent 7890 GC-MS system equipped with an HP-5MS Agilent column (30m × 0.250 mm × 0.25 μm) at the Lawrence Berkeley National Laboratory Catalysis Laboratory was used for GC-MS analysis. Nuclear magnetic resonance (NMR) spectra were collected on a Bruker AVQ-400 spectrometer at the University of California, Berkeley, College of Chemistry NMR Facility.

### Construction of Vectors and Strains

Multiple expression vectors for AibH1H2 were generated to enable straightforward protein synthesis or to ameliorate irreproducible protein yields or the ability to obtain high-quality single crystals. The specific utility and construction for each individual vector are described below and summarized in Table S3.

The His_6_-affinity-tagged pACYC-AibH1H2 plasmid was used to obtain single crystals of ^Fe^AibH1H2 and was constructed as follows. Codon-optimized genes of AibH1 (UniProt KB accession no. A0A402C2 V4) and AibH2 (UniProt KB accession no. A0A402C2Q3) were ordered from Twist Bioscience (Table S4). The genes were sequentially digested and ligated into the pACYC-GroEL/ES-TF vector (Addgene plasmid no. 83923) using standard protocols from New England Biolabs (NEB) and transformed into NEB Turbo chemically competent *E. coli* for amplification. The plasmid was extracted using a plasmid extraction kit (Biomiga), yielding the pACYC-AibH1H2 plasmid with chloramphenicol resistance. The complete sequence of both genes was confirmed by plasmid sequencing, and the pACYC-AibH1H2 was then transformed into BL21(DE3) competent *E. coli* for protein overexpression.

To increase protein expression yield, the AibH1 and AibH2 genes were moved to the pET-Duet vector with ampicillin resistance, allowing the genes to be coexpressed with the pACYC-GroEL/ES-TF plasmid (Addgene plasmid #83923), as the chaperone proteins GroEL/ES and TF promote efficient protein folding during heterologous expression. The pET-Duet-AibH1H2 plasmid was constructed following the same protocol as described above for the pACYC-AibH1H2 plasmid construction, except starting with the pET-Duet-1 vector (EMD Millipore Corp.), and the plasmid was confirmed by sequencing. The pET-Duet-AibH1H2 vector was transformed to *E. coli* BL21(DE3) cells pretransformed with pACYC-GroEL/ES-TF and made chemically competent following a previously reported protocol.^53^ This strain was used for all experiments, unless otherwise specified.

The AibH1H2-pET-Duet + GroEL/ES-TF strain was required to obtain a reasonable protein yield during heterologous expression. However, the protein grown from this strain did not reliably yield high-quality crystals of AibH1H2. Analysis of the AibH1H2 crystals that stemmed from the pACYC-AibH1H2 plasmid (8FUM and 8FUO) did not reveal detectable electron density prior to residue 28 on AibH1. Mass spectra of this protein in solution suggest an autocleavage event just before residue 18 that is not apparent for protein grown in the presence of the GroEL/ES-TF chaperone protein (Figure S15), and this cleavage is likely important for crystal packing. Therefore, to facilitate both high expression yields and crystal growth, a truncated pET-Duet-AibH1H2-Δ10-17 was generated by deletion of the corresponding base pairs of AibH1 from the pET-Duet-AibH1H2 plasmid (Table S4). The primers AibH1_Δ10-17-F (CTTATACTTTCAATCGAATCCAGGGGTACCGGATGAATTGG) and AibH1_Δ10-17-R (CTGGATTCGATTGAAAGTATAAGTTTTCGCCGCTGTGATG) were used in polymerase chain reaction (PCR). The NEB manufacturer PCR protocol for Phusion polymerase was followed with an annealing temperature of 75 °C. The resulting DNA was digested for 1 h with DpnI at 37 °C, and 5 μL of the PCR mixture was transformed directly into NEB Turbo *E. coli*. The plasmid was extracted, confirmed by DNA sequencing, and transformed to chemically competent *E. coli* BL21(DE3) cells pretransformed with pACYC-GroEL/ES-TF as described for pET-Duet-AibH1H2. This strain was used to generate the Mn/Fe-containing crystal structure (8FUN).

### Heterologous Protein Expression and Purification

All strains for protein overexpression were cultivated in either commercially available lysogeny broth (LB, Miller) or M9 minimal medium, as indicated above. M9 medium used herein contains Milli-Q water supplemented with 70 mM phosphate (KH_2_PO_4_/Na_2_HPO_4_), 8.6 mM NaCl, and 18.7 mM NH_4_Cl. The pH of these solutions was adjusted with NaOH to pH 7.4 and autoclaved, followed by the addition of 900 μM MgSO_4_, 175 μM CaCl_2_, and 0.375% glucose from filtered stock solutions. The medium was supplemented with 100 mg/L filtered ampicillin for strains containing pET-Duet-AibH1H2 and/or 34 mg/L chloramphenicol (dissolved in ethanol) for strains containing pACYC-AibH1H2 or pACYC-GroEL/ES-TF plasmid. Cultures were grown immersed in a water bath temperature set to 38 °C and vigorously aerated with HEPA-filtered compressed air. At an OD_600_ ≈ 0.6, the water bath temperature was decreased to 18 °C, and 0.125 mM divalent manganese or iron, in the form of MnCl_2_·4H_2_O or (NH_4_)_2_Fe(SO_4_)_2_(H_2_O)_6_, respectively, and 1 mM isopropyl β-d-1-thiogalactopyranoside (IPTG) were added immediately. The cultures were allowed to grow for 18–24 h before the cells were harvested by centrifugation at 5000 rpm for 5 min at 4 °C. The cell pellets were frozen at −80 °C until purification. All purification steps were performed at 4 °C. Thawed pellets from 12 L of growth media were resuspended in lysis buffer (200 mM NaCl, 50 mM HEPES pH 8.0) to a final volume of ∼60 mL and sonicated for 10 min of total pulse time (3 s pulse, 6 s off). For larger protein batches, sonication was successively performed in 60 mL fractions and then pooled following cell lysis. The lysate was centrifuged for 1 h at 12,000 rpm to remove cell debris, and the resulting supernatant was loaded onto a column equipped with Ni-nitriloacetic acid (NTA) resin (Thermo Fisher Scientific). The resin was subsequently washed with 100 mL of lysis buffer, washed further with 600–750 mL of wash buffer (lysis buffer + 25 mM imidazole), and eluted with 150 mL of elution buffer (lysis buffer + 250 mM imidazole). The protein was concentrated to <50 mL using an Amicon stirred cell (Millipore Sigma) equipped with a 30 kDa filter and subsequently buffer exchanged into 20 mM HEPES pH 8.0 via two rounds of dialysis against 4 L of buffer (one round was overnight and one for a minimum of 2 h). Protein purity was evaluated via sodium dodecyl sulfate–polyacrylamide gel electrophoresis (SDS-PAGE, Figure S16), and the metal content was determined using inductively coupled plasma optical emission spectroscopy (ICP-OES). Protein was stored at 4 °C until further experiments, except where further manipulation was required, as described below.

A final step to remove the His_6_-tag via TEV protease cleavage was required when the pET-Duet-AibH1H2-Δ10-17 plasmid was used. This afforded protein without the first 17 residues of AibH1, which appears to be important for crystallography (see the explanation above and Figure S15). The purified AibH1H2 protein was stirred in lysis buffer at a final concentration of 1 mg/mL with 2.5 mM dithiothreitol and 1 mg of TEV protease for every 10 mg of AibH1H2 overnight at 4 °C. The solution was passed down a Ni-NTA affinity column, and the flow-through collected and buffer exchanged to 20 mM HEPES pH 8.0 via dialysis as described above.

### Metal Chelation and Reconstitution

Protein that required further metal chelation and reconstitution was prepared the same way as described above, except the protein was dialyzed against 4 L of 20 mM HEPES pH 8.0 + 10 mM ethylenediaminetetraacetic acid (EDTA) for 2 h before the dialysis to 20 mM HEPES pH 8.0. Subsequently, the protein was degassed by equilibration in a Coy anaerobic chamber for at least 1 h. The sample was reduced by the addition of 6 mM sodium dithionite and 20 μM methyl-viologen and allowed to incubate at room temperature for 30 min. For Fe-grown protein, contaminating metals were chelated by treatment with 10 equiv (relative to the concentration of AibH1H2) of EDTA by addition of a degassed 200 mM EDTA stock and allowed to incubate for 1 h at room temperature. For Mn-grown protein, iron was selectively chelated by the addition of 10 equiv (relative to the concentration of AibH1H2) of ferrozine by the addition of degassed 100 mM ferrozine stock and incubation for 30 min at room temperature. *Exposure of protein samples to chelating molecules over longer periods of time results in enzyme preparations resistant to remetalation*. These partially chelated protein samples were then buffer exchanged in the Coy chamber by concentrating the protein to ∼5 mL using an Amicon stirred cell and filling with degassed 20 mM HEPES pH 8.0 up to 100 mL three times. The protein was then reconstituted by addition of 5 equiv of MnCl_2_·4H_2_O or (NH_4_)_2_Fe(SO_4_)_2_(H_2_O)_6_ to the Mn- or Fe-protein, respectively, and incubated anaerobically overnight at 4 °C. Excess metal was removed by repeating the buffer exchange in the Amicon stirred cell as described above. Metal content was determined by ICP-OES, and protein was stored at 4 °C until further experiments. The average yield of these combined metal chelation and reconstitution protocols is ∼70% (Fe-protein) and ∼60% (Mn-protein).

### Protein Mass Spectrometry

Protein samples were analyzed with an Agilent 1260 series liquid chromatograph (LC) connected in-line to an Agilent 6530 quadrupole time-of-flight (QTOF) mass spectrometer with an electrospray ionization source. The LC column used was a Proswift RP-4H column. Samples were analyzed using Agilent Mass Hunter software Qualitative Analysis Version B.10.0, Build 10.0 (Agilent Technologies Inc., 2020) (Figure S15).

### Determination of Protein Concentration

Protein concentration was estimated using the absorbance at 280 nm of protein samples in 20 mM HEPES (NanoDrop 2000c, Thermo Fisher Scientific). The extinction coefficients for AibH1 and AibH2 were predicted with the biochemical analysis tool on the Benchling Web site, and the expected extinction coefficient of AibH1H2 was calculated by taking the average of the constituent monomers (Table S3).

### Catalytic Activity Assays and GC-MS Analysis

Protein was degassed in a Coy chamber for at least 1 h prior to all of the enzymatic assays. The final concentration of protein was 25 μM in 100 μL reactions. One equivalent of the appropriate metal from a degassed 2.5 mM metal stock was added to the protein (20 mM HEPES, pH 8.0), and the resultant solution was incubated for 5 min anaerobically, followed by 5 min in air. The reaction was initiated by combining a premixed aerobic reaction mixture of 5 mM ascorbate (100 mM stock), 100 mM Aib (1 M stock), and 20 mM HEPES pH 8.0 (1 M stock) to the protein solution in a 1.5 mL Eppendorf tube. Time-dependent reactions (Figure 2c) were performed as described except with 200 mM AIB and 1 mM Ser as the internal standard added before initiating the reaction. The reaction tubes were incubated at 30 °C with the cap open and shaken at 300 rpm for 3 h (unless otherwise noted). ^1^H NMR spectroscopy (Figure S17) of the resulting filtered solutions was used to directly observe d-MeSer production. For quantitative analyses, the reaction products were silylated and analyzed by GC-MS. Reactions were quenched by the addition of 10 μL of 0.4 M trichloroacetic acid, followed by the addition of 2 μL of 50 mM serine to serve as an internal standard for GC-MS analysis. The protein precipitant was pelleted by centrifugation at 13,200 rpm for 5 min. A 90 μL amount of the supernatant was transferred to a 600 μL Eppendorf tube, frozen in liquid nitrogen, and lyophilized on a Schlenk line. The residue was combined with a 1:1 mixture of *N*-methyl-*N*-(trimethylsilyl)trifluoroacetamide and acetonitrile (50 μL each) and incubated at 70 °C for 30 min. The debris was pelleted by centrifugation for 5 min, and 1 μL of the supernatant was analyzed by GC-MS (Figures S2 and S3). The temperature was held at 80 °C for 2 min, then ramped up to 300 °C at 15 °C/min, and finally held at 300 °C for 10 min. d-MeSer production was quantified using the extracted ion chromatogram (EIC) at *m*/*z* = 218 divided by the EIC at *m*/*z* = 218 of Ser, which served as an internal standard. The yields were determined by comparison to a calibration curve of 100 μL of d-MeSer standard subjected to the same experimental conditions and workup as the activity assays. A new calibration curve was prepared at the beginning of each new experiment. Reactions were analyzed with ^18^O_2_ as described above (Figure S10), except all reaction components were degassed and mixed in an anaerobic Coy chamber and added to a J-Young tube. They were then attached to a Schlenk line equipped with ^18^O_2_, which was then added to the J-Young tube. The sample was quenched inside of the Coy chamber with 0.4 M trichloroacetic acid, and all subsequent workup and analysis were performed as described.

### X-ray Crystallography

Crystals of all proteins were obtained by sitting-drop vapor diffusion at room temperature in an anaerobic Coy chamber. All solutions and crystal trays (Hampton) were degassed overnight prior to use. The protein was degassed in the Coy chamber for at least 1 h and subsequently incubated with any additional metals for at least 15 min prior to crystallization. Each reservoir was filled with 200 μL of precipitant solution, and 2 μL of the solution was added to the well containing 2 μL of 6 mg/mL protein. Experimental crystal growth conditions are summarized in Table S5. Good quality crystals generally appeared after 2–4 weeks of maturation. The crystal for the Tris-bound ^Fe^AibH1H2 structure was transferred to a well containing 2 μL of the precipitant solution plus 25% PEG400 with 200 μL of this solution in the reservoir and incubated for 14 h for cryoprotection prior to harvesting and freezing in liquid N_2_. All other crystals were harvested and frozen in liquid N_2_ directly. X-ray crystallographic data were collected at the Advanced Light Source (ALS) or the Stanford Synchrotron Radiation Lightsource (SSRL), with the specific beamlines and the energy of data collection tabulated in Table S5. Diffraction data were processed using iMosfilm followed by scaling and merging using Aimless.^54,55^ Phasing was performed using Phaser-MR via molecular replacement with PDB ID: 6M2I as the initial search model. The final models were generated by refinement in phenix.refine and manual modeling with COOT. Electron density maps were generated in Phenix, and PyMol was used for the generation of all figures. Anomalous difference maps were created with Phenix and iron-specific anomalous DANO maps were created using the ScaleAndMerge and Fobs_minus_Fobs routines within Phenix.^56^ No map sharpening algorithms were utilized in the analysis of the anomalous dispersion data.

### Electron Paramagnetic Resonance Spectroscopy

EPR studies were performed in the CalEPR center at the University of California, Davis. X-band continuous-wave EPR spectra were recorded on a Bruker Biospin EleXsys E500 spectrometer equipped with a super high Q resonator (ER4122SHQE) in perpendicular mode. Cryogenic temperatures were achieved and controlled using an ESR900 liquid helium cryostat with a temperature controller (Oxford Instrument ITC503). Spectrometer settings were as follows: conversion time, 60 ms; modulation amplitude, 0.8 mT; modulation frequency, 100 kHz. The samples in 1 M Tris were collected at 15 K and 20 mW power, and the CHES samples were collected at 12 K and 63.25 mW power. EPR spectra were simulated in Matlab with Easyspin 5.2.28 toolbox.^57^

The naturally abundant Fe (^NA^Fe) EPR samples were prepared as follows. Samples of as-isolated ^Mn^AibH1H2 (in 20 mM HEPES pH 8.0) were degassed by equilibration in a Coy chamber for at least 1 h. One equivalent of (NH_4_)_2_Fe(SO_4_)_2_(H_2_O)_6_ was added to the protein and incubated at room temperature for 5 min prior to exposure to air. The samples were then buffer exchanged into the appropriate air-saturated buffer (1 M Tris or 20 mM CHES pH 8.9) via five rounds of concentration and dilution in 0.5 mL of an Amicon centrifugal unit with a 30 kDa membrane (Millipore). The sample with 100 mM AIB was prepared as described above except that the buffer contained 20 mM CHES and 1 M AIB (pH 8.9), and the sample was subsequently diluted 10× with 20 mM CHES pH 8.9 to yield a final concentration of 100 mM AIB in 20 mM CHES pH 8.9. After incubation in air for 1 h, the samples were frozen in liquid N_2_ for EPR analysis. Final protein concentrations were 250 μM AibH1H2 (in CHES) or 271 μM AibH1H2 (in Tris).

For the ^57^Fe sample in 1 M Tris, a 50 mM ^57^FeCl_2_ stock solution was prepared anaerobically in 100 mM HCl and frozen at −80 °C until use. Upon thawing in the anaerobic Coy chamber, ^57^FeCl_2_ was diluted 10× to 5 mM using 100 mM HEPES pH 8.0. One equivalent of this 5 mM ^57^FeCl_2_ stock was added to mildly chelated ^Mn*^AibH1H2 (see metal chelation protocol) to minimize contamination of ^NA^Fe. The sample was oxidized and buffer exchanged, as described above, prior to freezing in liquid N_2_.

### Bioinformatic Analyses

A BLAST search of the AibH2 amino acid sequence (Uniprot: A0A402C2Q3) on the UniProt Reference Proteome database was used to identify all homologues displaying >25% sequence identity. The resultant sequences were aligned using MUSCLE^58^ within the UGene software package.^59^ Sequences lacking one or more of the aligned amino acid residues found to coordinate the Mn/Fe cofactor (25D, 27H, 211H, 265E, 337D, and 340H in AibH2) were removed, leaving 555 unique proteins. The genome neighborhoods (Figure 5b) of these proteins were downloaded and evaluated using the Enzyme Function Initiative suite of online tools and visualized using Gene Graphics.^60,61^ To correlate the amino acid conservation with predicted 3D structures, 255 sequences were randomly eliminated, and the resultant 300 sequences were used to generate Figure 5d using the default algorithm parameters on the ConSurf Web site.^62^

All resultant 555 amino acid sequences (and AibH2) are classified to the protein family (Pfam) PF04909. To evaluate their evolutionary relatedness to other biochemically characterized enzymes within this family, these sequences were aligned alongside the 30 SwissProt-annotated PF04909 sequences and the sequence for PtmU3. The resultant multisequence alignment was manually curated via the elimination of all aligned amino acid positions bearing >50% gaps. This alignment was analyzed using the NGPhylogeny suite of tools^63^ to create the neighbor-joining, unrooted phylogenetic tree in Figure 5a. PhyML was used to determine the best evolutionary model (LG substitution, empirical equilibrium frequencies, Gamma shape estimated at 1.513), and the tree was constructed with the AIC statistical criterion and NNI tree topology. Branch support values employed the aBayes approximation^64^ and revealed a high level of confidence across the majority of the tree. The Interactive Tree of Life was used for graphic generation.^65^

## Supporting Information Available

X-ray diffraction data, MeSer product characterization, EPR simulations and parameters (PDF)

## Accession Codes

All structures were validated and deposited in the Protein Data Bank (PDB) under the following accession numbers: 8FUL, AibH1H2 expressed from lysogeny broth; 8FUM, Fe-metalated AibH1H2 with bound Tris; 8FUN, Mn/Fe-metalated AibH1H2; 8FUO, Fe-metalated AibH1H2.
