# Supplementary material for: Enzymatic Hydroxylation of Aliphatic C–H Bonds by a Mn/Fe Cofactor
Source: J Am Chem Soc. 2023 Jul 20;145(30):16526–37. doi: 10.1021/jacs.3c03419 (PMC10401708; doi:10.1021/jacs.3c03419)
Supplement: Supplementary file 1 — ja3c03419_si_001.pdf [file ja3c03419_si_001.pdf]

Supporting Information for

Enzymatic Hydroxylation of Aliphatic C-H Bonds by a Mn/Fe Cofactor

Magan M. Powell[1], Guodong Rao[2], R. David Britt[2], Jonathan Rittle[1*]

[1]Department of Chemistry, University of California, Berkeley; Berkeley, CA 94720, USA

[2]Department of Chemistry, University of California, Davis; Davis, CA 95616, USA

*Corresponding author. Email: rittle@berkeley.edu

Contents

Supplementary Text	Page
	2

Supplementary Figures	Page
Figure S1	3
Figure S2	4
Figure S3	5
Figure S4	6
Figure S5	7
Figure S6	8
Figure S7	9
Figure S8	10
Figure S9	11
Figure S10	12
Figure S11	13
Figure S12	14
Figure S13	15
Figure S14	16
Figure S15	17
Figure S16	18
Figure S17	19

Supplementary Tables	Page
Table S1	20
Table S2	21
Table S3	22
Table S4	23
Table S5	24
Table S6	25

Supplementary Schemes	Page
Scheme S1	26

Supplementary Text

Attempts were made to preclude metal binding in AibH1 by substituting one or more residues at/near Site 0 but resulted exclusively in protein that was insoluble. A simple E267A mutant of AibH1 was found to result in insoluble protein. Reasoning that the loss of the metal ion might influence the overall protein fold of AibH1 (and hence its ability to form adequate protein-protein interactions with AibH2), we used Rosetta-packer to design mutants intended to furnish soluble, metal-free AibH1. The attempted substitutions to AibH1 listed below similarly resulted in insoluble protein following over expression in *E. coli*:

1. E267A, D36N, H10L, D340G, H315L
2. E267A, D36N, H10L
3. E267A, D36N, H10L, D340G, H315L, H72F

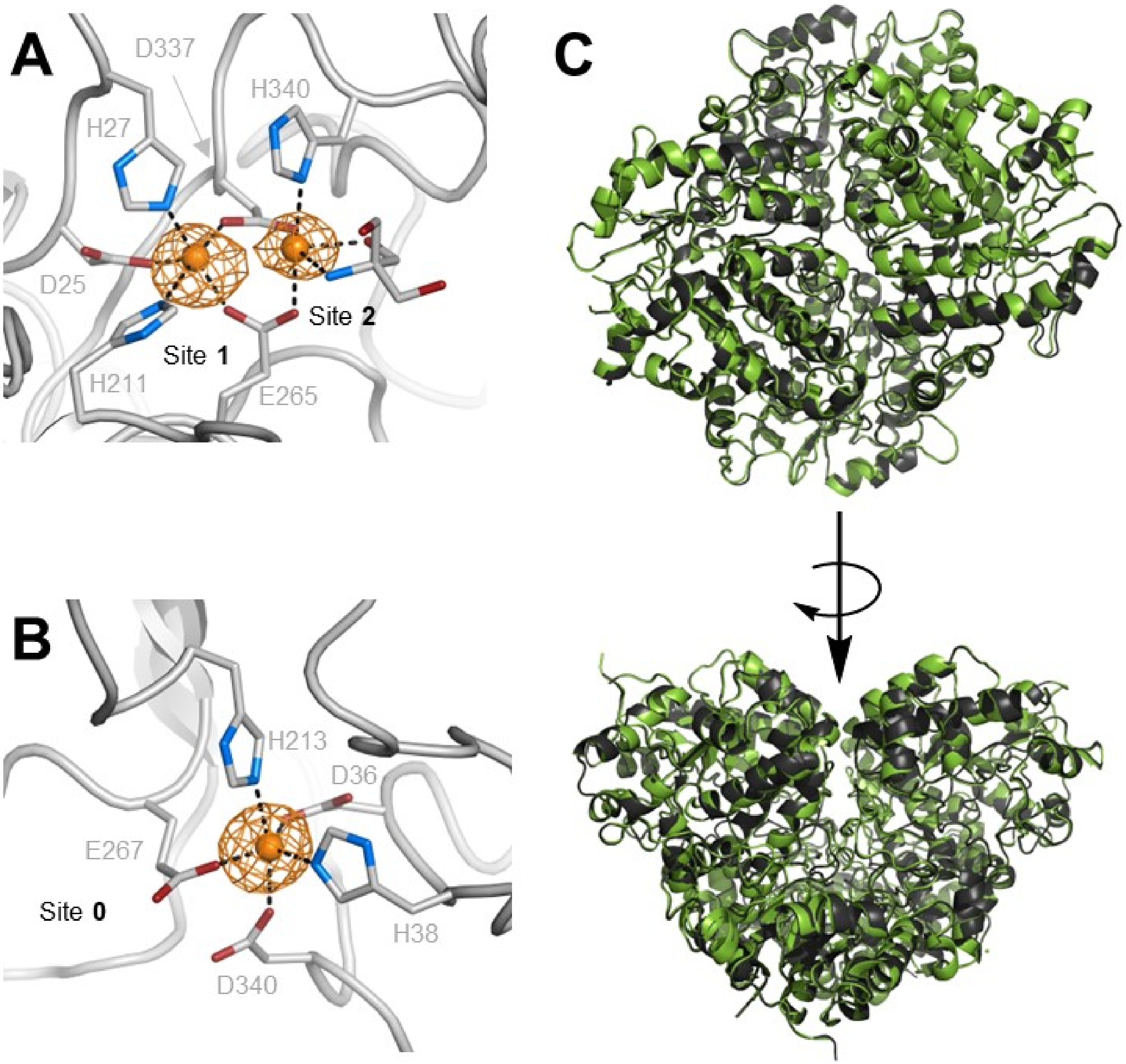

Figure S1. Anomalous dispersion density maps (contoured at 11σ) at the (A) dinuclear AibH2 site and the (B) mononuclear AibH1 site. X-ray diffraction data were collected at the Fe K-edge (7132 eV) on a crystal of ^{Fe}AibH1H2 (8FUO). (C) Structural overlay of ^{LB}AibH1H2 (green, 8FUL) and ^{Fe}AibH1H2 (black) (RMSD = 0.34 Å).

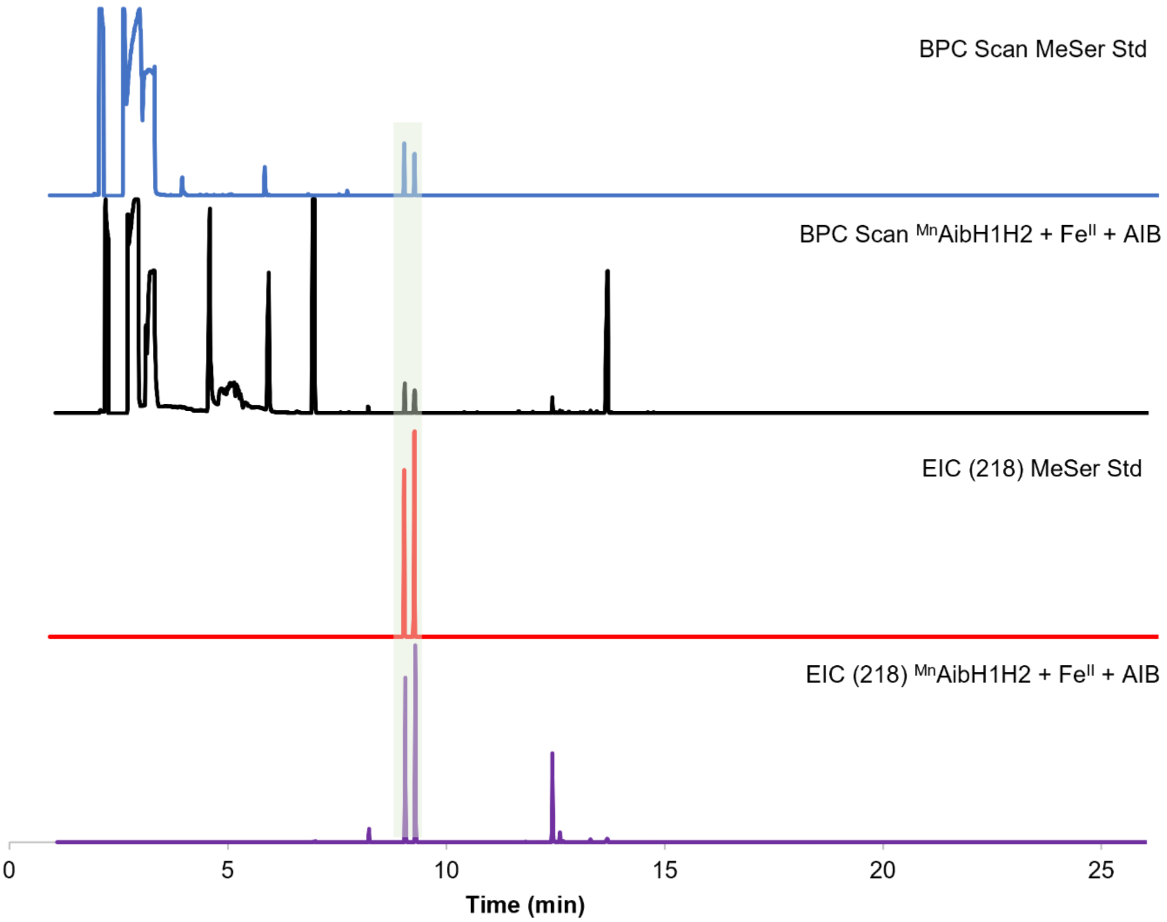

Figure S2. Complete base peak chromatogram (BPC) and the complete extracted ion chromatogram (EIC) filtered at m/z = 218 for a representative enzymatic assay of ^{Mn}AibH1H2 and 1 equiv Fe^{II} and a MeSer standard. The peaks for MeSer (elution time 9.3 minutes) and the internal standard serine (9.1 minutes) are highlighted in green.

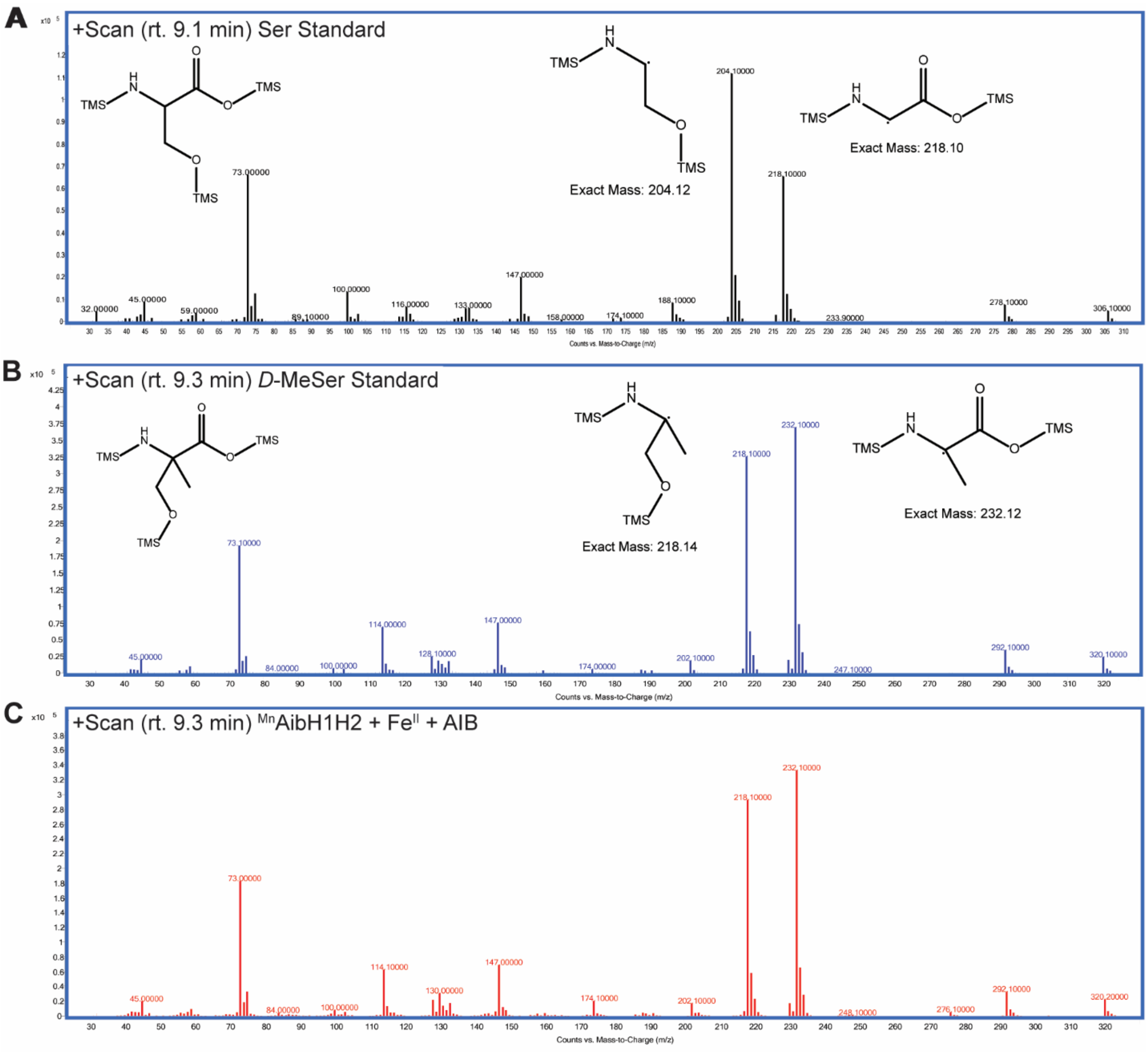

Figure S3. Mass spectra for the silylated products of (A) the Ser internal standard, (B) the *D*-MeSer standard, and (C) the product of the enzymatic reaction of ^{Mn}AibH1H2 and 1 equiv Fe^{II}. The retention times, indicated at the top of each mass spectrum, correspond to the chromatograms in Figure S2. Structures of the silylated standards are shown on the left of each mass spectrum, and the ionization fragment giving rise to the two most abundant mass peaks are shown next to the respective peak. Because Ser and *D*-MeSer both have a m/z = 218 fragment, 218 is used to filter all chromatograms for quantification.

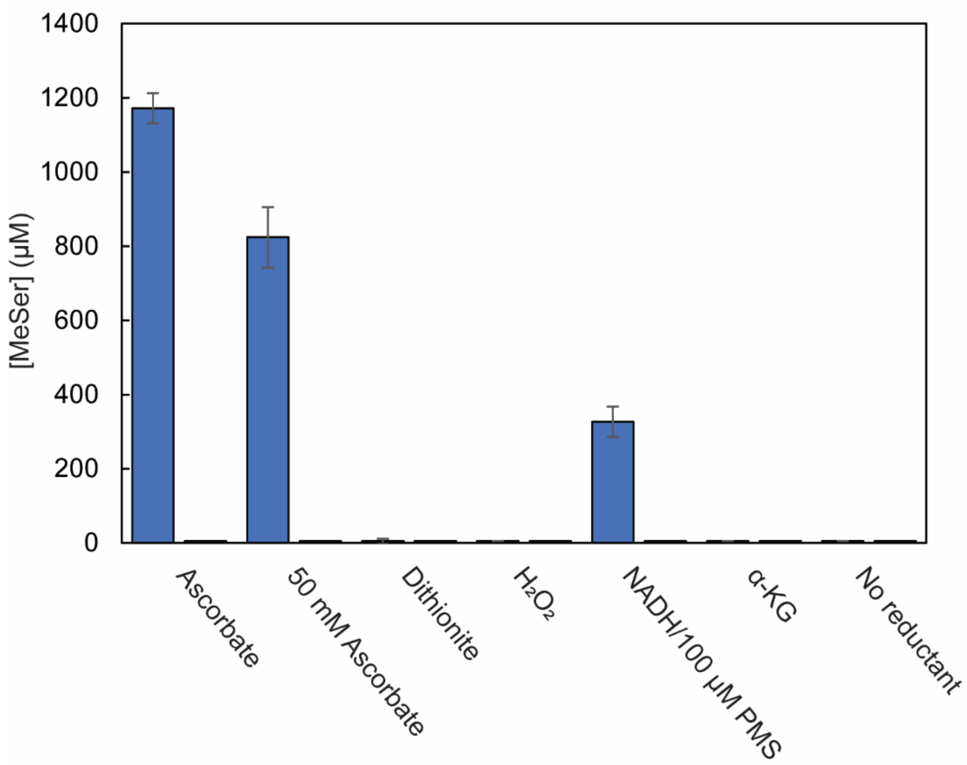

Figure S4. *D*-MeSer production from enzymatic assays performed either in the presence of 25 μM ^{Fe}AibH1H2 (red) or 25 μM ^{Mn}AibH1H2 (blue), an additional equivalent of Fe^{II}, and the listed small molecules to serve as reducing equivalents. Final concentrations of all small molecules were 5 mM unless otherwise indicated. Water was added in place of a reductant where indicated. PMS = phenazine methosulfate, α-KG = alpha-ketoglutarate. Each bar is the mean of three independent trials, and the error bars represent the standard deviation in observed *D*-MeSer yield. In all experiments with ^{Fe}AibH1H2, no *D*-MeSer was observed above the detection limit (~25 μM).

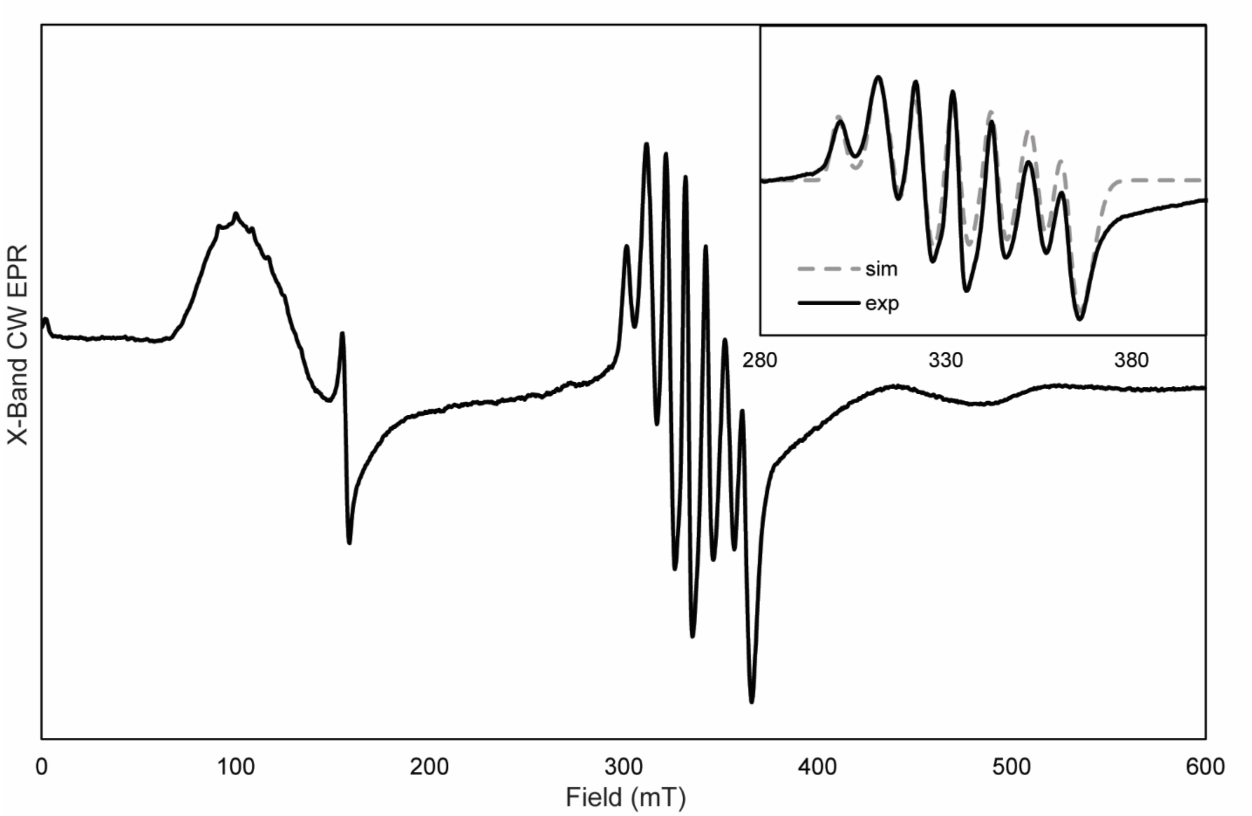

Figure S5. X-band CW EPR spectrum of 270 μM ^{LB}AibH1H2 in 20 mM HEPES buffer, pH 7.5. Spectrum collected at 5 K and 2 mW power. The inset is a simulation of the feature centered around 350 mT, with $\boldsymbol{g}$- (2.04 1.97 1.92) and ^{55}Mn hyperfine ($\boldsymbol{A}_{\mathrm{Mn}}$ = [300 260 230] MHz) tensors consistent with an S = ½ Mn(III)-Fe(III) species. This sample was prepared in a different buffer and pH than all others in this study, and therefore these simulation parameters are not directly comparable to other spectra. The broad, low-field feature centered at ~100 mT exhibits ^{55}Mn hyperfine coupling. We tentatively ascribe this feature to a mononuclear Mn^{II} species possessing a large zero-field splitting, similar to that of Mn-sequestering solute-binding proteins.[1] It is not presently clear if this spectral feature stems from a Mn^{II} ion in Site 0 of AibH1 or a partially metalated AibH2. Further simulation and analysis of this spectrum would require high-field EPR measurements. The feature at g = 4.3 is from non-specifically bound Fe.

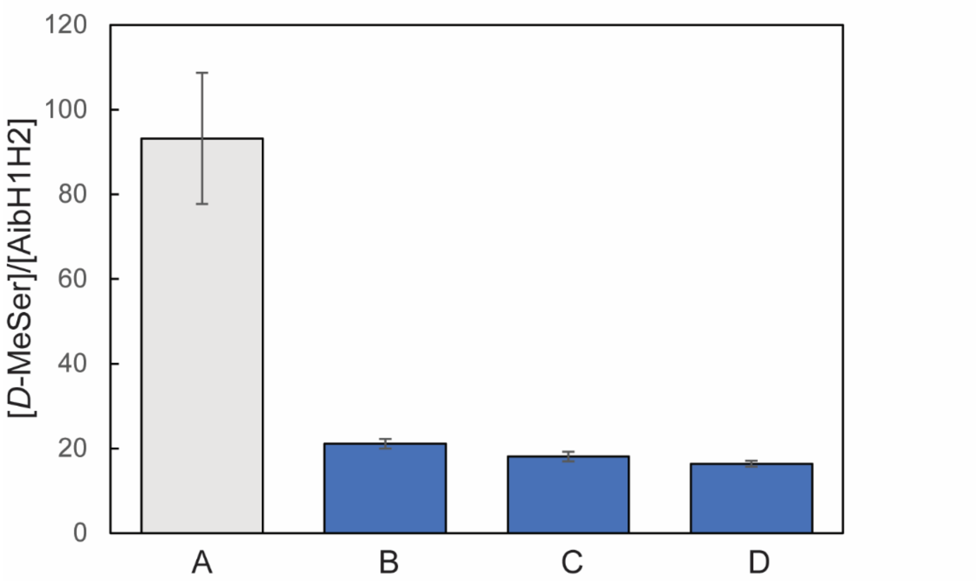

Figure S6. Enzymatic activity of ^{Mn}AibH1H2 + 1 equiv Fe^{II} (gray) and the same sample of ^{Mn}AibH1H2 after the chelation protocol described in the main text (aerobic EDTA treatment followed by anaerobic ferrozine treatment). Apo protein was remetallated by the addition of 2 equiv Mn^{II} and 1 equiv Fe^{II} after a 5 min incubation (B), addition of 2 equiv Mn^{II} and 1 equiv Fe^{II} pre-mixed before protein addition (C), or addition of 1:1 mixture of Mn^{II}:Fe^{II} with a total metal content of 3 equivalents per AibH1H2 (D). Although these are the same batch of protein, AibH1H2 is resistant to complete remetallation, resulting in lower enzymatic turnover. This demonstrates why the activity for ^{Mn*}AibH1H2 (Fig. 2C and Table 1 entry 4) is lower than ^{Mn}AibH1H2 (Fig. 2B and Table 1 entry 3).

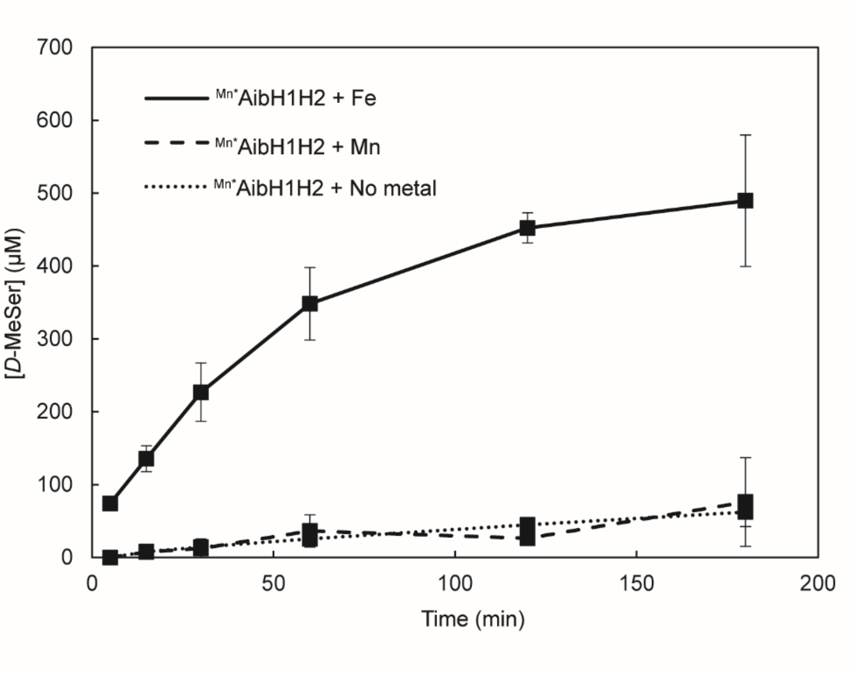

Figure S7. Reactivity of ^{Mn*}AibH1H2 with one additional equivalent of Fe (solid line), Mn (dashed line), or an equal volume of water (dotted line). Data represents an average of three technical replicates, and the error bars are the standard deviation. The activity when either Mn or no metal is added is nearly identical and significantly lower than that of ^{Mn*}AibH1H2 plus Fe. We predict the low activity in both cases might be due to adventitious Fe in the reaction vessel.

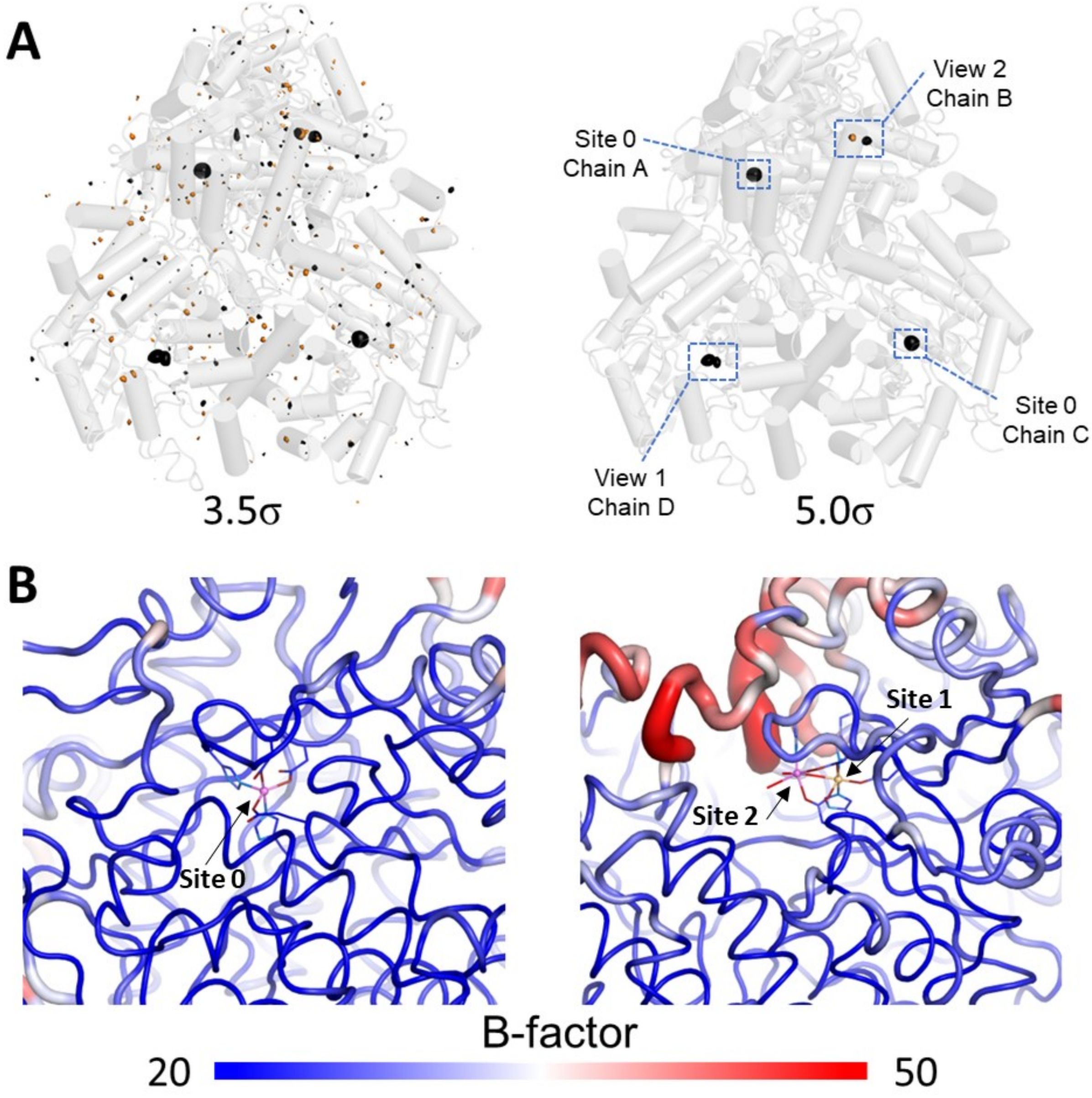

Figure S8. Observable metal ion content determined by anomalous diffraction throughout the entire crystal lattice and local peptide flexibility surrounding the different metallocofactors. (A) Anomalous difference density map at the Mn K-edge (6550 eV, black surface) and Fe-specific dual-wavelength anomalous density difference map (7092 and 7132 eV) each drawn at 3.5σ (left) and 5.0σ (right) for structure 8FUN. The labels correspond to the specific metal binding sites discussed in the main text. At 3.5 σ, the anomalous response is sufficiently noisy to preclude identification of discrete metal sites, but at higher thresholds, the only observable metal ions are found within the mono- and dinuclear active sites of the protein. (B) Cartoon representations of the peptide environments surrounding Site 0 (left) and Sites 1 and 2 of View 2 (right). The local color and radius of the cartoon representation reflect the average experimental thermal parameters (B factor) of each amino acid.

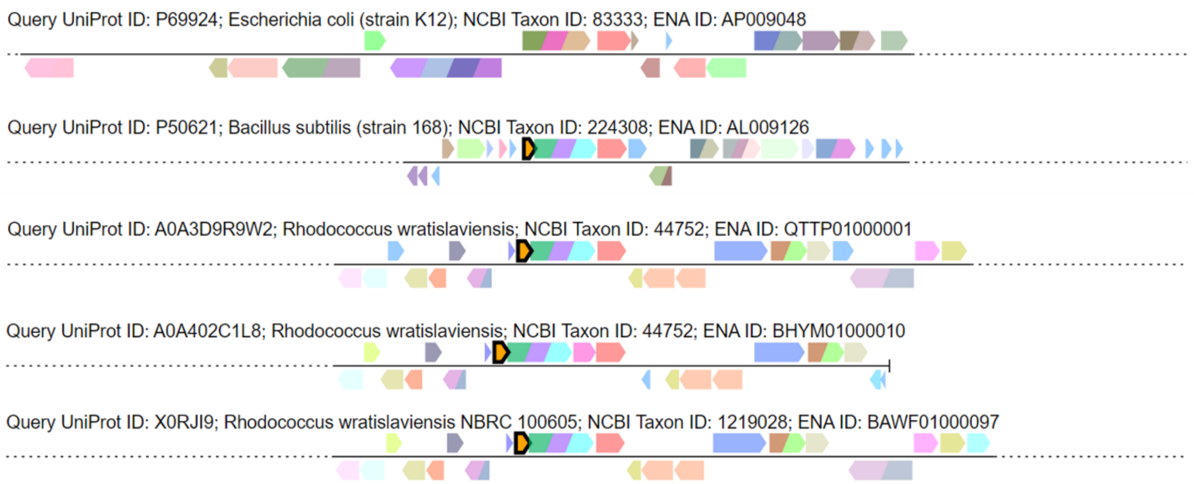

Figure S9. Genome neighborhood diagrams (GNDs)[2] of the genes encoding class I ribonucleotide reductase (RNR) and its surrounding operon in several organisms. All RNRs in *R. wratislaviensis* are adjacent to the *nrdI* gene (bolded in orange), which has previously been suggested as a putative indicator of manganese-dependent, class Ib RNR.[3-6] GNDs of biochemically characterized RNRs from *E. coli* (class Ia) and *B. subtilis* (class Ib) are shown as examples of experimentally verified iron-dependent and manganese-dependent RNRs without and with the *nrdI* gene, respectively.[7,8]

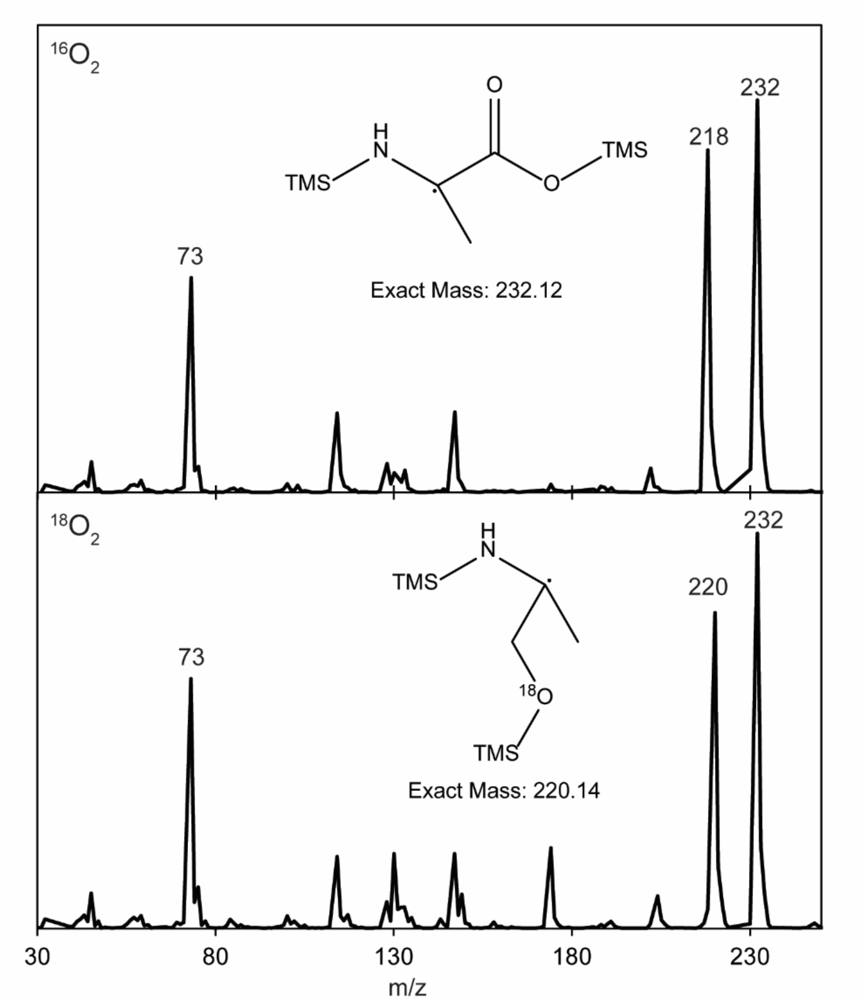

Figure S10. Mass spectrum of natural abundance silylated *D*-MeSer (top) and the product of a reaction of ^{Mn}AibH1H2 + Fe with $^{18}O_2$ (bottom). The reaction was performed as described in the main text, except all reaction components were degassed and mixed in an anaerobic Coy chamber and added to a J-Young tube. It was then attached to a Schlenk line equipped with $^{18}O_2$, which was added to the J-Young tube. The sample was shaken at 300 rpm at 30 °C for 3 hours and then quenched inside of the Coy chamber with 0.4 M trichloroacetic acid. Subsequent workup and analysis were performed as described in the main text. The m/z = 232 fragment does not include the hydroxyl group (top schematic) and the exact mass is retained in the $^{18}O_2$ experiment. The m/z = 218 fragment does include the hydroxyl group (bottom schematic) and the mass of this fragment increases by 2 units to m/z = 220 upon $^{18}O_2$ exposure.

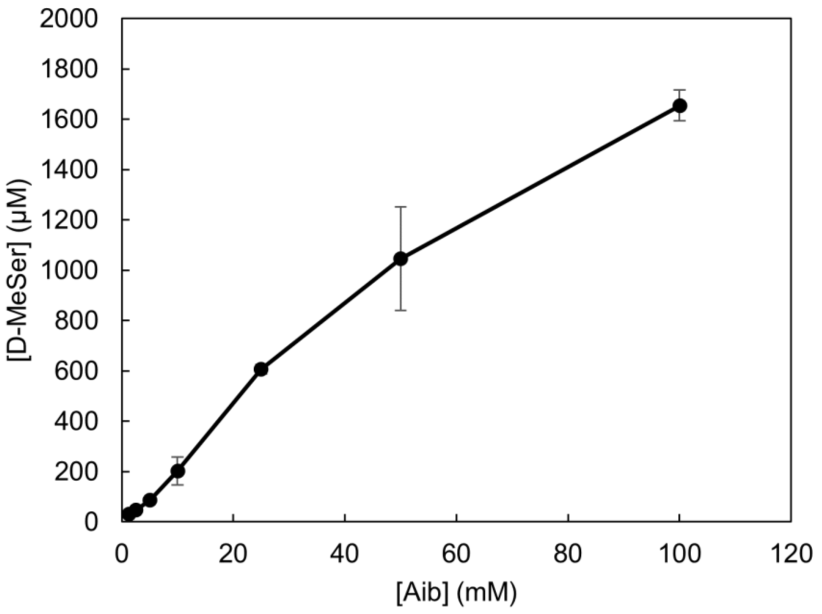

Figure S11. Enzymatic activity of 25 µM ^{Mn}AibH1H1 + 1 equiv Fe^{II} with variable [AIB]. Assays were performed as described in the main text except for the variable [AIB].

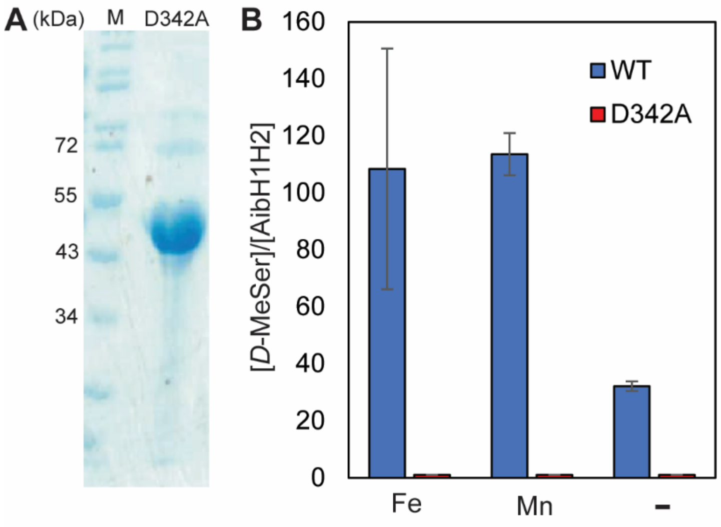

C

	Metal/heterodimer			
	Fe	Mn	Ni	Zn
D342A	0.06	1.85	0.58	0.13
WT	0.25	2.34	0	0

Figure S12. Biochemical characterization of AibH1H2 with the secondary sphere substitution D342A on AibH2. AibH1H2-D342A was purified as described for the wild type (WT) protein in the main text without any further metal chelation, and SDS-PAGE analysis (A) verified highly soluble protein. The D342A variant yielded negligible *D*-MeSer product during enzymatic assays with the addition of one equivalent of Fe, Mn, or an equal volume of water (denoted by "-") to the reaction (B) despite its ability to acquire metals consistent with the WT protein as indicated by ICP-OES analysis (C). Values of the enzymatic assays are reported as an average of three technical replicates, and the error is the standard deviation. ICP-OES values less than the limit of detection of the instrument (0.01 ppm) are reported as zero. The ICP-OES values are an average of three injections of a single sample with an error of <0.01 ppm.

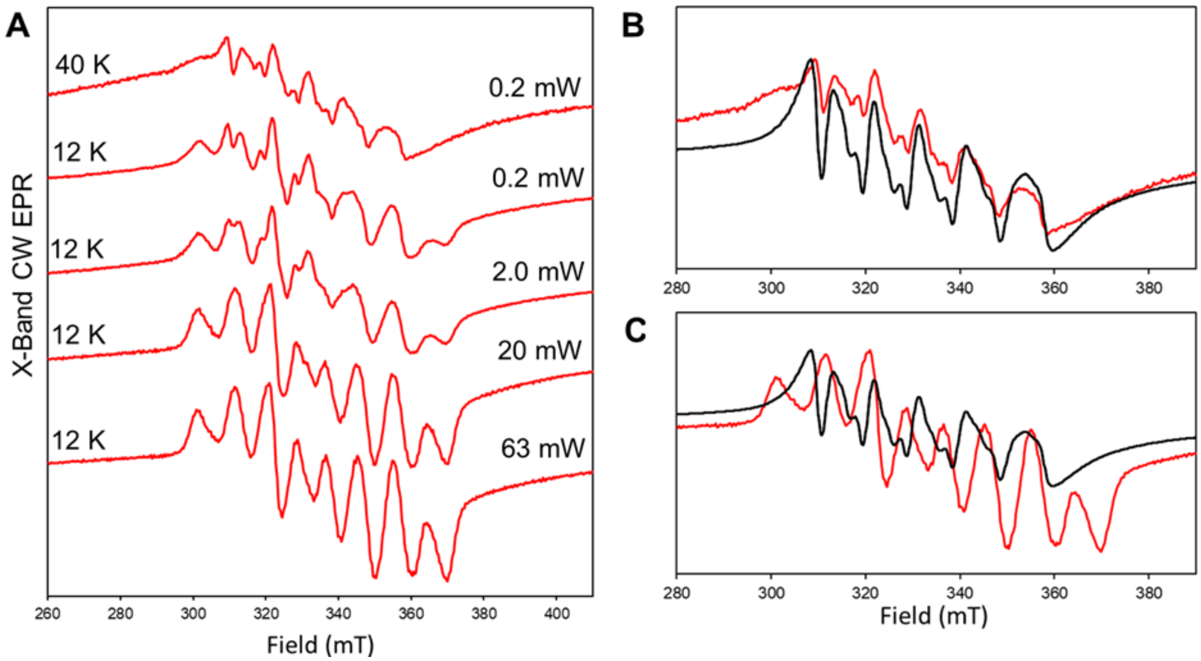

Figure S13. (**A**) Normalized X-band CW EPR spectra of ^{Mn}AibH1H2 in 20 mM CHES pH 8.9 (red) collected at the listed powers and temperatures. Overlays of the X-band CW EPR spectrum of an aqueous 250 µM $MnCl_2$ standard (black, 5K, 2 mW) and **(B)** ^{Mn}AibH1H2 at 40 K and 0.2 mW (red) or **(C)** ^{Mn}AibH1H2 at 12 K and 63 mW (red). These data are meant to show the progressive removal of the Mn^{II} signal upon increasing the power and decreasing the temperature. By 12 K and 63 mW, the sample does not appear to have any contributing Mn^{II} signal. The spectra in this figure stem from a different batch of ^{Mn}AibH1H2 than those in the main text. Batch-to-batch variations in small molecule content (e.g., buffer, salt concentrations) that may coordinate near or at the cofactor are likely the cause of the different spectral shape of the main text Figure 4c (top) and Figure S13a (12 K, 63 mW) despite being collected under the same experimental conditions.

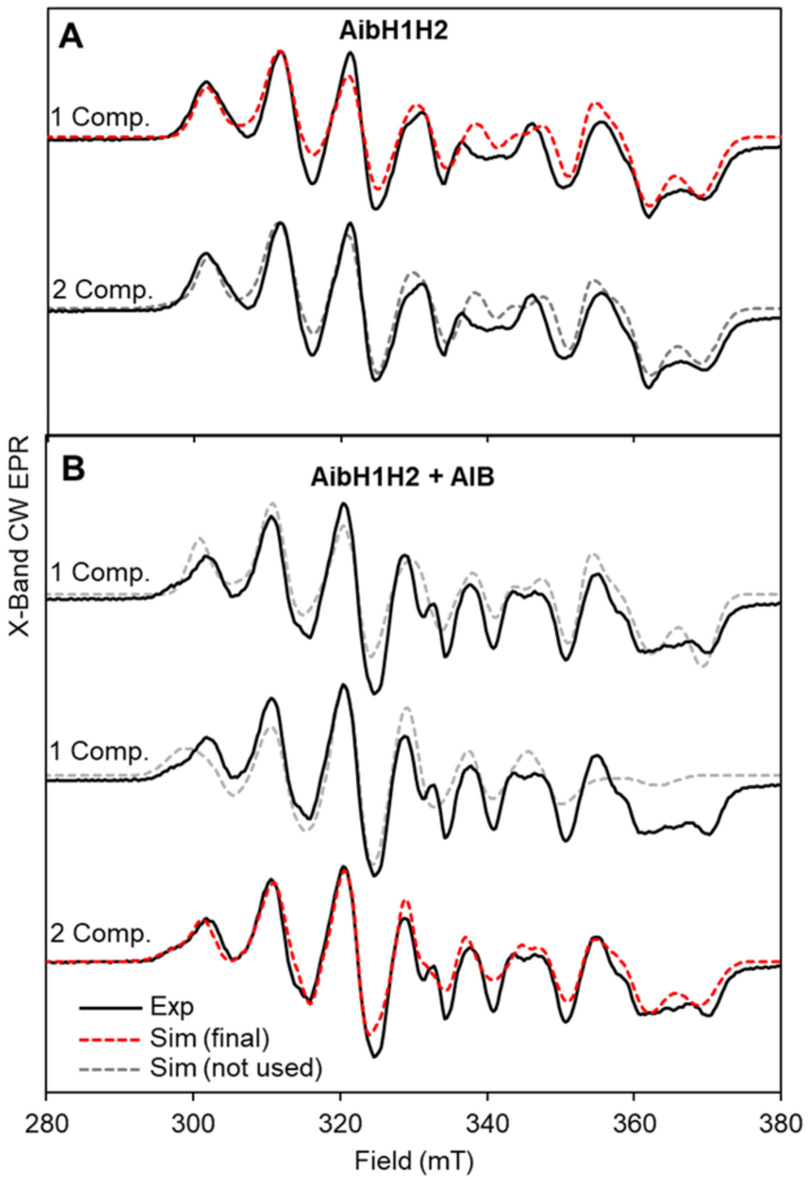

Figure S14. Different simulations of X-band EPR spectra of ^{Mn}AibH1H2 in 20 mM CHES pH 8.9 without (**A**) and with (**B**) 100 mM AIB. Experimental spectra are shown as solid black lines, the best simulations chosen for the main text are in red dashed lines, and attempted simulations that were not used are in gray dashed lines. For the sample without AIB (**A**), the fit is similar with a single- or multiple-component simulation, and therefore a single-component simulation was chosen. For the sample with AIB (**B**), simulations with only one component failed to simultaneously model the new feature around 297 mT and the high-field features.

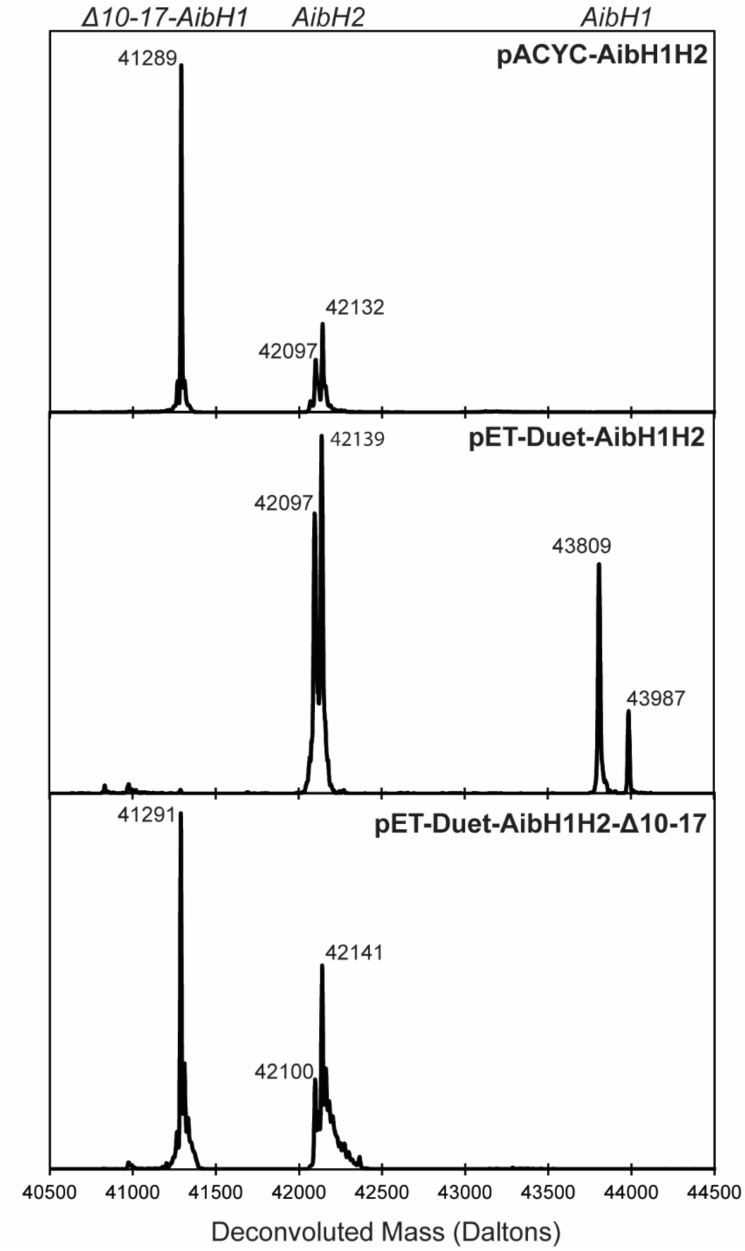

Figure S15. Quadrupole time of flight mass spectrometry (QTOF-MS) of different AibH1H2 preparations. AibH1H2 grown from the pACYC vector yields AibH1 with a mass of 41.3 kDa instead of the expected mass of 43.9 kDa, indicating spontaneous cleavage of the protein. For all experiments except three crystal structures (8FUO, 8FUM, and 8FUN), AibH1H2 grown in the pET-Duet vector (with the GroEL/ES-TF chaperone protein) was used because the chaperone protein enables higher expression yields, and this results in masses close to the theoretically expected mass (see Table S3). However, this protein did not crystallize as reliably as the auto-cleaved protein grown from the pACYC plasmid. Therefore, the pET-Duet-AibH1H2-Δ10-17 strain was constructed to afford large quantities of protein that could also be reliably crystallized.

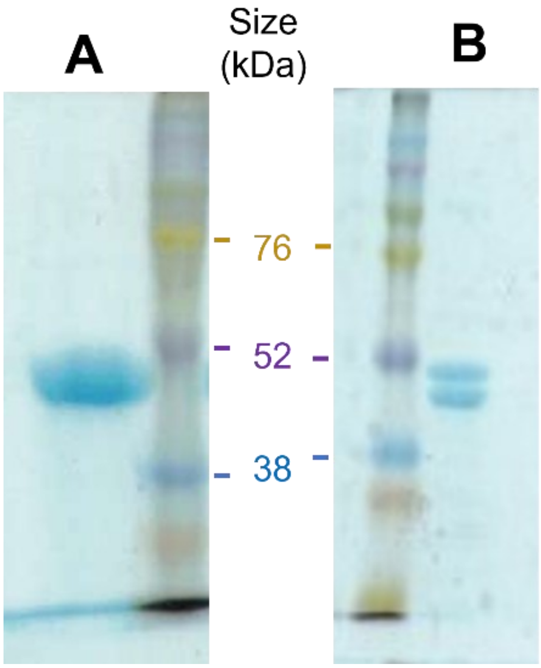

Figure S16. Representative SDS-PAGE gels of (A) full-length AibH1H2 and (B) AibH1H2 with a Δ10-17 truncation on AibH1.

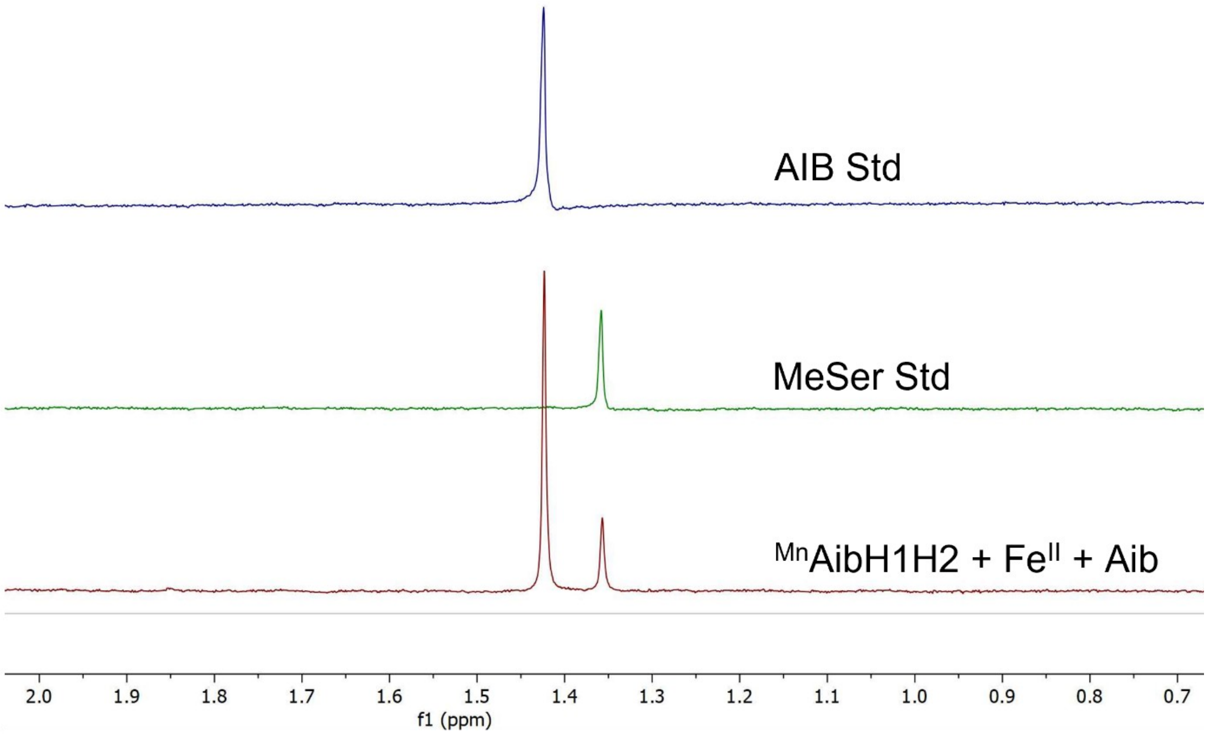

Figure S17. ^{1}H NMR (D_2O, 400 MHz, 22 ºC) spectroscopic characterization of enzymatic reaction products. Enzymatic reactions were performed as described in the main text using 500 µL total reaction volume and employing 200 µM ^{Mn}AibH1H2, 200 µM Fe^{II} metal, 5 mM ascorbate and 5 mM AIB. Following 3 h of aerobic agitation, the protein was removed by centrifugation in a 30 kDa Amicon® centrifugal unit. A 400 µL aliquot of the resultant flowthrough was added to 100 µL of D_2O for ^{1}H NMR spectroscopic analysis.

Table S1. Anomalous difference peak heights for structure 8FUN reported in σ values.

	Site 0	Site 1	Site 2	^{35}Cys(S) AibH1	^{116}Cys(S) AibH1	^{107}Cys(S) AibH2	^{131}Cys(S) AibH2	^{328}Cys(S) AibH2
Chain A								
Mn Edge	**18.8**	-	-	4.5	3.4	-	-	-
Fe Difference	2.2	-	-	1.6	2.3	-	-	-
Chain B								
Mn Edge	-	5.3	**8.1**	-	-	2.2	2.8	2.6
Fe Difference	-	**7.4**	2.6	-	-	2.1	2.9	3
Chain C								
Mn Edge	**17.9**	-	-	2.2	3.1	-	-	-
Fe Difference	<2	-	-	2.1	2.4	-	-	-
Chain D								
Mn Edge	-	**12.4**	**7**	-	-	2.7	3.7	4
Fe Difference	-	3.5	<2	-	-	2.3	2.1	3.2

Note: Boldened values reflect our atomic assignments. Peak heights at the five cysteine sulfur atoms present in AibH1H2 are included to gauge the intrinsic noise level in this dataset.

		g_1	g_2	g_3	g_{iso} [a]	g_{aniso} [b]	Mn^{III} A_1	A_2	A_3	A_{iso}	A_{aniso}
^{NA}Fe (1 M Tris)		2.033	2.029	2.024	2.029	0.007	303	245	375	308	101
^{57}Fe (1 M Tris)		2.033	2.029	2.024	2.029	0.007	303	245	375	308	101
No Aib (20 mM CHES)		2.042	1.984	1.928	1.985	0.086	298	259	242	266	48
100 mM Aib (20 mM CHES)	Sys1[c]	2.042	1.984	1.928	1.985	0.086	298	259	242	266	48
	Sys2	2.055	2.044	2.020	2.039	0.030	264	199	385	283	154

	Fe^{III} A_1	A_2	A_3	A_{iso}	A_{aniso}
^{57}Fe (1 M Tris)	-65	-66	-68	-66	2.5

[a]The isotropic g- and A- terms are an average of the individual values. [b]The anisotropy in the g- and A- tensors is given as the difference between the unique component and the average of the other two components. [c]The ratio of Sys1:Sys2 is 3.63:0.51

Table S2. Simulation parameters for the EPR spectra discussed in the main text.

Table S3. Summary of plasmids used in this study.

Name	Molecular Weight (kDa)			ε_{280} (M^{-1} cm^{-1})	Application
	**AibH1**	**AibH2**	**AibH1H2**		
pET-Duet-AibH1H2	43.9	42.2	86.1	72365	EPR, catalytic activity assays, crystal structure of ^{LB}AibH1H2 (PDB: 8FUL)
pACYC-AibH1H2	43.9	42.2	86.1	72365	^{Fe}AibH1H2 crystals (PDB: 8FUM and 8FUO)
pET-Duet-AibH1H2-Δ10-17	41.3[a]	42.2	83.5	71620	^{Mn*}AibH1H2 + Fe crystal (PDB: 8FUN)

[a]Size after removal of the His_6-tag by TEV cleavage

Table S4. Gene blocks (5' to 3') used for AibH1H2 plasmid construction.

AibH1

ATGC**CCATGG**GC**CATCATCATCATCATCAC**AGCGGC**GAAAACTTATACTTTCAATCCGGT**GGCATGGTTGCGC
CCACCTCGAATCCAGGGGTACCGGATGAATTGGACGGTGTACCTGCGGTCGTGGACTGCGACGTACACGCGGT
TTTGCCGTCCCCTCATTCTCTGATTCCATATTTGGACGAGTACTGGGCCGACCAGCTTGTCGCACAGTTAGCTCC
TACGTATGAACCTAACTACCATCCGCGCGGATCCGCAATAGCACAACACTCAGACGCGAGTGTTGATGAGAACG
GCCGTGCGGCCACCACGGCTGAGAACTTGGTGAAGGACGTATTCGCCGACGGCTTTACGGACTTTGCTGTGGT
CAACTGCCTTTACGGCGTGCAACAAATACACCAACCTCGGCGTGAGATGGCACATGCTCGGGCTTTGAACCACT
GGATAGCGAATGAGTGGCTGGATAAAGACGATCGTCTGAGAGCCAGTATCGTGGTTCCCCAAGGGAGCCCTCG
GGCTGCCGCTGAGGAGATCGATTTTTGGTCTGGAGACAAGAGATTCGTACAAGTATTGCTGTTAGGGCAGAGCG
AACTGCTTTACGGAAGAGAAATCAATTGGCCCATCTGGGAAGCGGCAGAAGCTGCCGGGCTTCCCGTAACTTTA
CATATTGGTGGCGTTTTTCGTCAGGCTCCAACTAGCGTGGGATGGCCCGCATCACATTTGGAATGGTACGTGGG
ACAGCAGAGTAATATCGAGGCCCAGCTGAACTCGATCATTAGCGAAGGGATTCTTCAAAAATTTCCAAAAACAAA
GATCTTATTAAGTGAACTTGGCTTCAACTGGCTTCCACCGTTTATGTGGAAATTTGACAAATTATGGAAAAGCTAT
AGACCCGATATTCCTTGGGTTCAGGAATCGCCTTTGGAATTGATACGCGAGCACGTTCGGGTTACCACCAGTCC
AAGCGATGGCGCAGAGGAGGCAGGCCGCCTTGACTCTATCGTGGACCGGTTAGGAAGTGACCGCATGTTAGTT
TACAGTTCTGATTATCCCCACAAGCACCACTCAGGTCCAAGAGACATTGAAAATGGGACCCACAGCCCTGAATTA
TTGGACCGGATATACCGTCGGAACGCCTTCGATCTTTACAACCTTGTAGTGCCCTCCCCCGGAAAAGTGGGTTA
A**AAGCTT**ATGC

AibH2

GGAATTCCATATGACGATAATCGAGCATGGGTCCCTGGGTACATTACCGGCGCCAAGTGTCACCACCGGGATC
GTCGACGCGGATATCCATCCAGTACCACAGGACGGCGCGCTTGAGCCATATCTGGATGATCGTTGGAAAAAACA
CATCAGAGAATACGGAGTCCGGACGACCACAGGCCTGCAGTTTATTTCAGAGTACCCACAAATGTATGGTGGGG
CAATGAGAGCAGACGCCTGGCCTGAATCGGGGTATCCTGGGAGCGACCGTGAGCTTCTGCGGACACAATTGTT
GGACAAACACAATATACAACTGGGGGTGTTGCAGTGCCTTGCCCCCGGCGGGCAAACCCTGAATCCGGCTGGA
CAGGCCTTAAACCAAGAACTTGCCGCTGCCCTTTGTCGTGCTACTAATGACTGGCAATTGGAGCACCTTGTGTAC
CCCGACCCACGGATGAGAGCTGCAATCCCTGTGACTTTTGAGACCCCTGACTACGCAGTCGCTGAAATAGAACG
TGTGGGAGCAGATCCAGGTGTCGTTGCCGTTTTAGGGACGAGCAAAACGTTGGAGCCTCTGGGAAGTCGCAAG
TACTGGCCGATCTACGAGGCGTCCGTCGCGCAGAATCTTCCGATACAGTTCCACTTGTCGCAAGGCGGGGGAC
ACGCTAATACAGGAACGGGATGGACCTCATATCATACGGAATATCACACAGGACATGTTCAATCTTTTCAATCGC
AGTTACTGAGTCTTGTATTATCAGGCACTTTCGACCGCTTTCCAACCCTTAAAGTTATGTTCGTGGAGGGTAATGT
GGCTCATTTCGCTCCACTTATCCAACGCATGGATTATACTTGGGAGACGCTTCGCGGAGAGCTGCCAGACCTGC
AGCGTAAGCCATCTGAGTACATACGTGATCACATTTGGGCGAGCACCCAGCCCATAGATGAGCCTGAAAAGCCG
GAGCACTTAGCGGAGTTATTAGAAGAGTTCTGCGGGGACAATGTCGTTTTCGCAACAGATTACCCCCACTTTGAT
TTCGATGACCCTGAGACTGCGTTTCCACGCTCGTTTCCTGTCGACCTTAGAGATAAGATCTTACGCGGAAATGGT
ATGCGCTTTTTTGGCGTAACGAACCAGGCTGATTAA**CTCGAG**ATGCG

Note: Restriction enzyme recognition sequences are colored red and underlined, linker bases and the stop codon (TAA) are colored red, the 6x-His tag is underlined and bolded, and the TEV protease recognition sequence is bolded. Base pairs that were removed to generate pET-Duet-AibH1H2-Δ10-17 are highlighted in gray.

Table S5. Summary of crystallization conditions in this study.

Crystal	Plasmid	Beamline	Energy (eV)	Metal[a]	Precipitant
8FUN	pET-Duet-AibH1H2-Δ17-24	ALS 8.2.2	6550, 7132, 7092	$(NH_4)_2Fe(SO_4)_2(H_2O)_6$	0.16 M $MgCl_2$, 0.08 M Tis-HCl pH 8.7, 24% PEG4000, 50 mM AIB, 20% glycerol
8FUM	pACYC-AibH1H2	SSRL 9-2	12658	$MnCl_2{\cdot}4H_2O$	0.16 M $MgCl_2$, 0.08 M Tis-HCl pH 8.7, 20% PEG4000, 50 mM AIB
8FUO	pACYC-AibH1H2	SSRL 9-2	7132	*none*	0.16 M $MgCl_2$, 0.08 M Tis-HCl pH 8.7, 20% PEG4000
8FUL	pET-Duet-AibH1H2	ALS 5.0.2	12658	*none*	0.16 M $MgCl_2$, 0.08 M HEPES pH 8.5, 20% PEG4000, 20% glycerol, 10 mM DT

[a]2 equivalents of metal added relative to [AibH1H2]

	^{Fe}AibH1H2	^{LB}AibH1H2	^{Fe}AibH1H2 + Tris	^{Mn*}AibH1H2 + 2 equiv Fe^{II}	^{Mn*}AibH1H2 + 2 equiv Fe^{II}	^{Mn*}AibH1H2 + 2 equiv Fe^{II}
**PDB ID**	8FUO	8FUL	8FUM	8FUN		
**Data Collection**						
Wavelength	1.738 Å	0.979 Å	0.979 Å	1.738 Å	1.748 Å	1.893 Å
Resolution range (Å)	79.74 - 2.43 (2.52 - 2.43)	47.28 - 2.29 (2.33 - 2.29)	45.58 - 1.48 (1.53 - 1.48)	46.0 - 2.24 (2.29 - 2.24)	46.0 - 2.24 (2.29 - 2.24)	46.0 - 2.50 (2.58 - 2.50)
Space group	I 2 2 2	I 1 2 1	I 1 2 1	I 2 2 2	I 2 2 2	I 2 2 2
Unit cell	84.78 150.57 234.72 90 90 90	82.77 231.40 145.00 90 92.17 90	82.59 232.64 147.64 90 92.77 90	84.14 148.92 234.09 90 90 90	84.19 148.83 233.87 90 90 90	84.22 148.83 233.89 90 90 90
Total reflections	650852 (44054)	373023 (18655)	1102416 (117933)	800957 (32071)	800785 (30431)	615729 (47432)
Unique reflections	55038 (5260)	119390 (11703)	431064 (42532)	70480 (6547)	70089 (3953)	51127 (4361)
Multiplicity	11.8 (10.3)	3.1 (3.1)	2.8 (2.6)	11.4 (8.0)	11.4 (7.7)	12.0 (10.9)
Completeness (%)	96.7 (93.9)	97.9 (96.1)	93.72 (92.58)	99.3 (93.46)	98.9 (87.5)	99.8 (99.6)
Mean I/sigma(I)	18.3 (3.0)	8.1 (4.1)	7.2 (1.2)	5.2 (1.8)	5.6 (1.8)	7.7 (3.0)
Wilson B-factor	49.9	46.15	12.7	25.25		
R-merge	0.085 (0.738)	0.08 (0.202)	0.085 (0.818)	0.486 (1.504)	0.487 (1.566)	0.267 (0.805)
R-meas	0.089 (0.777)	0.108 (0.281)	0.105 (1.027)	0.527 (0.809)	0.534 (1.799)	0.290 (0.886)
R-pim	0.026 (0.236)	0.061 (0.154)	0.060 (0.608)	0.154 (0.585)	0.153 (0.626)	0.082 (0.263)
CC1/2	0.999 (0.810)	0.989 (0.915)	0.996 (0.355)	0.901 (0.293)	0.916 (0.337)	0.972 (0.753)
Anom Completeness	97.3 (93.4)	n/a	n/a	98.6 (84.8)	98.4 (83.2)	99.7 (99.2)
Anom Multiplicity	6.1 (5.3)	n/s	n/a	5.8 (4.2)	5.8 (4.0)	6.2 (5.5)
**Refinement**						
Reflections used in refinement	55032 (5259)	119391 (11704)	430996 (42524)	70481 (6548)		
Reflections used for R-free	2000 (191)	2000 (191)	1414 (140)	2000 (186)		
R-work	0.188 (0.264)	0.1894 (0.3813)	0.159 (0.253)	0.1774 (0.2656)		
R-free	0.250 (0.347)	0.2563 (0.4716)	0.184 (0.250)	0.1979 (0.2837)		
Number of non-hydrogen atoms	11658	23276	25133	12070		
macromolecules	11347	22658	22812	11433		
ligands	14	25	180	16		
solvent	297	593	2141	621		
Protein residues	1430	2852	2859	1436		
RMS(bonds)	0.009	0.009	0.009	0.012		
RMS(angles)	1.3	1.32	1.36	1.4		
Ramachandran favored (%)	94.51	94.98	96.96	95.94		
Ramachandran allowed (%)	5.07	4.84	2.79	3.57		
Ramachandran outliers (%)	0.42	0.18	0.25	0.49		
Rotamer outliers (%)	3.49	2.33	1.03	1.48		
Clashscore	5.22	5.69	3.24	4.01		
Average B-factor	52.91	48.49	17.46	27.6		
macromolecules	53.21	48.6	16.9	27.59		
ligands	58.9	62.06	35.09	38.64		
solvent	41.15	43.37	21.87	27.64		

Table S6: X-ray data collection and refinement statistics. Numbers in parenthesis correspond to statistics of the highest resolution shell.

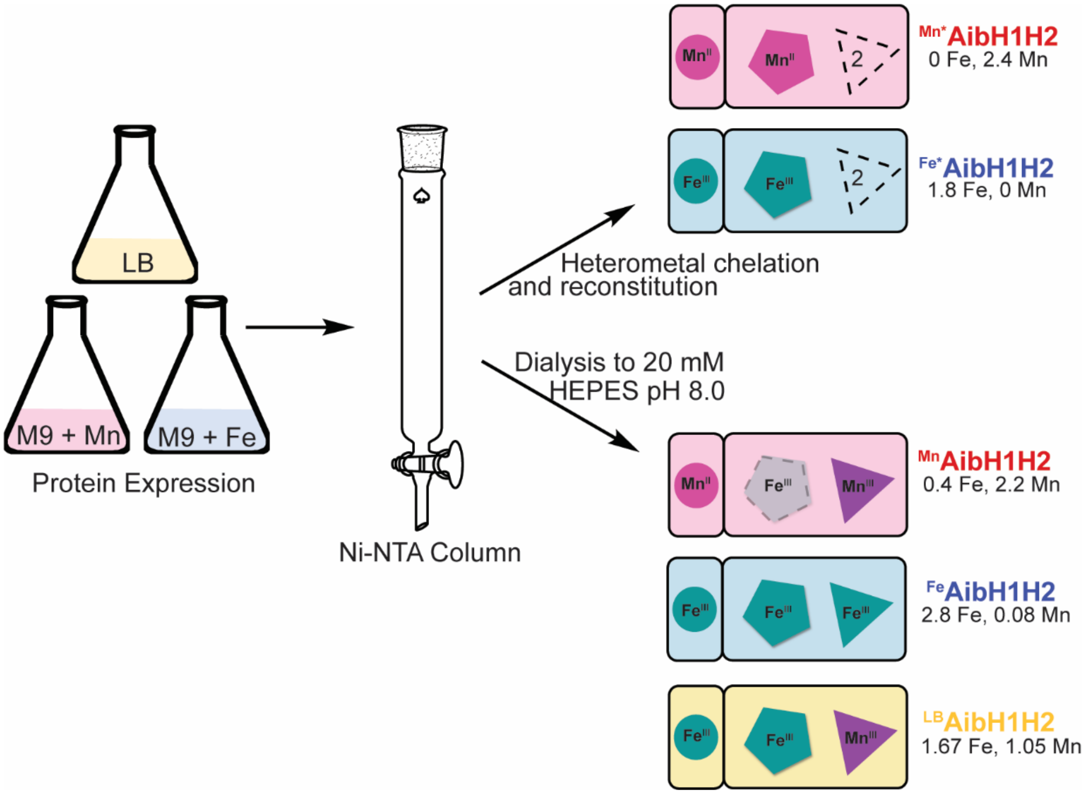

Scheme S1. Visual representation of different protein preparations discussed in the main text. Average ICP values of each preparation method are shown next to the protein graphic. An additional equivalent of divalent metal was subsequently added to an aliquot of the protein at the time of the experiment, as indicated for each experiment in the main text.

Supplemental References

1. Hadley, R. C.; Gagnon, D. M.; Brophy, M. B.; Gu, Y.; Nakashige, T. G.; Britt, R. D.; Nolan, E. M. Biochemical and Spectroscopic Observation of Mn(II) Sequestration from Bacterial Mn(II) Transport Machinery Calprotectin. *J. Am. Chem. Soc.* **2017**, *140* (1), 110-113. https://doi.org/10.1021/jacs.7b11207.
2. Zallot, R.; Oberg, N.; Gerlt, J. A. The EFI Web Resource for Genomic Enzymology Tools: Leveraging Protein, Genome, and Metagenome Databases to Discover Novel Enzymes and Metabolic pathways. *Biochemistry* **2019** *58* (41), 4169-4182. https://doi.org/10.1021/acs.biochem.9b00735
3. Roca, I.; Torrents, E.; Sahlin, M.; Gibert, I.; Sjöberg, B.-M. NrdI Essentiality for Class Ib Ribonucleotide Reduction in *Streptococcus Pyogenes*. *J Bacteriol* **2008**, *190* (14), 4849–4858. https://doi.org/10.1128/JB.00185-08.
4. Boal, A. K.; Cotruvo, J. A.; Stubbe, J.; Rosenzweig, A. C. Structural Basis for Activation of Class Ib Ribonucleotide Reductase. *Science* **2010**, *329* (5998), 1526–1530. https://doi.org/10.1126/science.1190187.
5. Cotruvo, J. A.; Stubbe, J. NrdI, a Flavodoxin Involved in Maintenance of the Diferric-Tyrosyl Radical Cofactor in Escherichia Coli Class Ib Ribonucleotide Reductase. *Proc. Natl. Acad. Sci.* **2008**, *105* (38), 14383–14388. https://doi.org/10.1073/pnas.0807348105.
6. Cotruvo, J. A. J.; Stubbe, J. An Active Dimanganese(III)−Tyrosyl Radical Cofactor in Escherichia Coli Class Ib Ribonucleotide Reductase. *Biochemistry* **2010**, *49* (6), 1297–1309. https://doi.org/10.1021/bi902106n.
7. Salowe, S. P.; Stubbe, J. Cloning, Overproduction, and Purification of the B2 Subunit of Ribonucleoside-Diphosphate Reductase. *J Bacteriol* **1986**, *165* (2), 363–366. https://doi.org/10.1128/jb.165.2.363-366.1986.
8. Zhang, Y.; Stubbe, J. *Bacillus Subtilis* Class Ib Ribonucleotide Reductase Is a Dimanganese(III)-Tyrosyl Radical Enzyme. *Biochemistry* **2011**, *50* (25), 5615–5623. https://doi.org/10.1021/bi200348q.
